# Supplementary material for: A specific prelimbic-nucleus accumbens pathway controls resilience versus vulnerability to food addiction
Source: Nat Commun. 2020 Feb 7;11:782. doi: 10.1038/s41467-020-14458-y (PMC7005839; doi:10.1038/s41467-020-14458-y)
Supplement: Supplementary file 1 — Supplementary Information [file 41467_2020_14458_MOESM1_ESM.pdf]

Supplementary Information

A specific prelimbic-nucleus accumbens pathway controls resilience versus vulnerability to food addiction

Domingo-Rodriguez et al.

Supplementary Figures

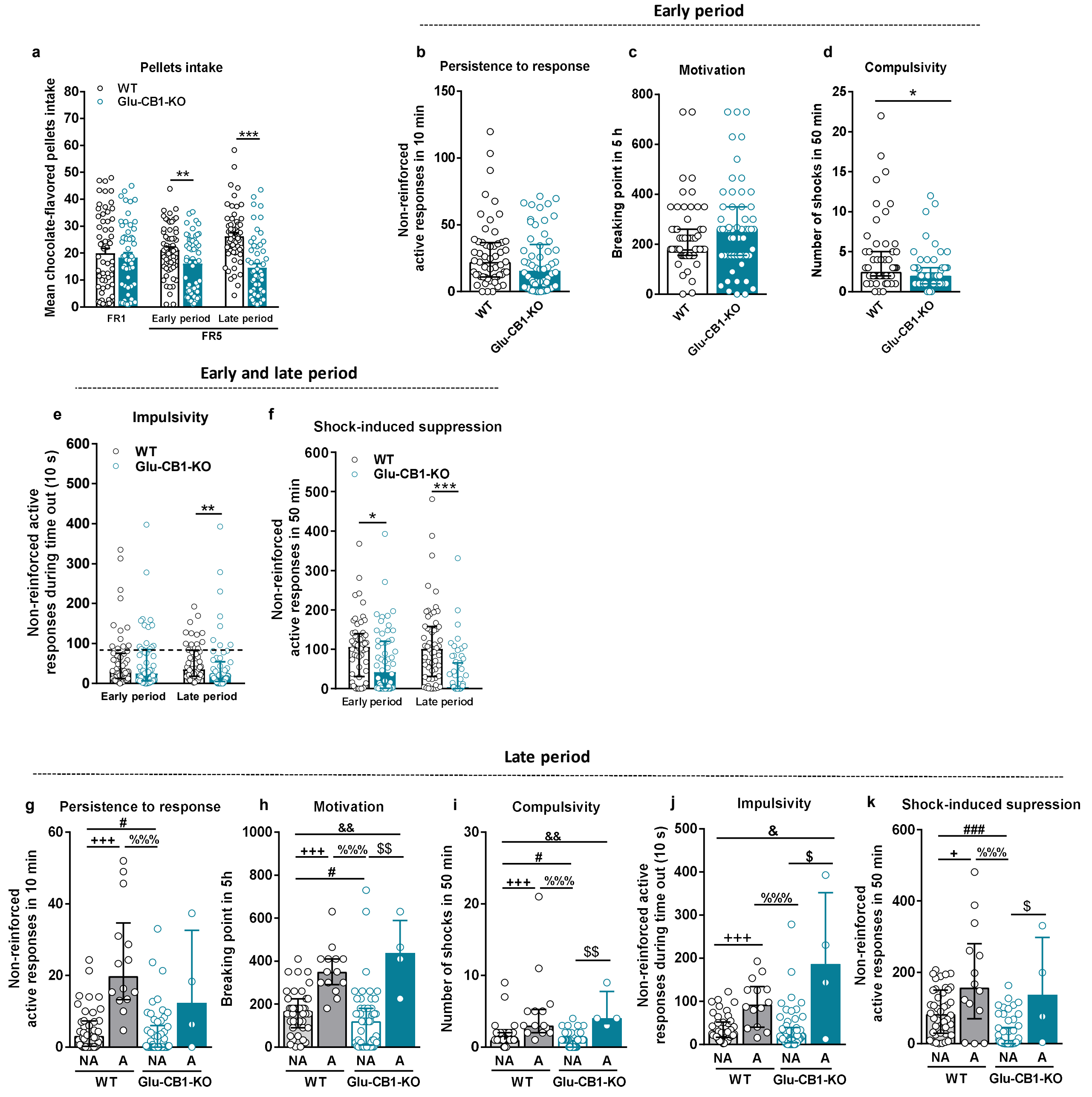

Supplementary Figure 1. Glu-CB1-KO mice display resilience to food addiction. a, Mean of chocolate flavored-pellets intake in FR1 and FR5 comparing early and late periods (mean ± S.E.M, t-test, **P<0.01, ***P<0.001). **b-d,** Behavioral tests of the three addiction-

like criteria represented by individual values with the median and the interquartile range in both genotypes at the early period. **b,** Persistence to response. Total number of non-reinforced active responses during three consecutive daily 10-min of pellet free period. **c,** Motivation. Breaking point achieved in 5 h of PR schedule. The breaking point refers to the maximal effort that an animal is willing to do to earn one pellet. **d,** Compulsivity. Number of shocks that mice received in 50 min in the shock test in which each pellet delivery was associated with a footshock (0.18 mA) (U Mann-Whitney, *P<0.05). **e-f,** Behavioral tests of impulsivity and shock-induced suppression in the early and late period represented by individual values with the median and the interquartile range. **e,** Impulsivity. Number of non-reinforced active lever-presses during three consecutive daily time out (10 s) after each pellet delivery (U Mann-Whitney, **P<0.01). **f,** Shock-induced suppression. Number of non-reinforced active responses in 50 min in the following session after the shock test with the same discriminative stimulus (grid floor) as shock test in which pressing the active lever had no consequences: no shock, no chocolate-flavored pellets and no cue-light (U Mann-Whitney, *P<0.05, ***P<0.001). **g-k,** Behavioral tests of the three addiction-like criteria, impulsivity and shock-induced suppression in the late period represented by individual values and bars with median and the interquartile range for the four groups classified as addicted (A) and non-addicted (NA) mice in both genotypes. **g,** Persistence to response. **h,** Motivation. **i,** Compulsivity. **j,** Impulsivity. **k,** Shock-induced suppression (U Mann-Whitney, +P<0.05, +++P<0.001 WT NA vs WT A, #P<0.05, ###P<0.001 WT NA vs Glu-CB1-KO NA, &P<0.05, &&P<0.01 WT NA vs Glu-CB1-KO A, %%%P<0.001 WT A vs Glu-CB1-KO NA, $P<0.05, $$P<0.01 Glu-CB1-KO NA vs Glu-CB1-KO A; n=56 for WT mice and n=58 for Glu-CB1-KO mice; statistical details are included in Supplementary Table 1).

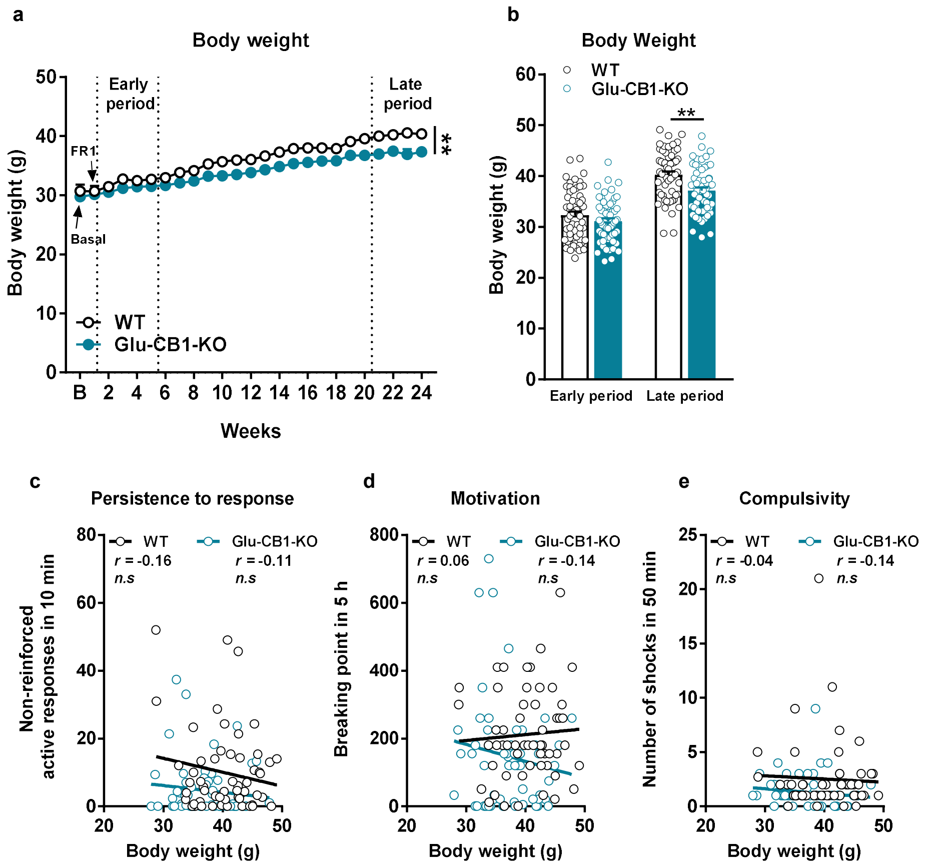

Supplementary Figure 2. Decreased body weight in Glu-CB1-KO is not a predisposing factor in food addiction-like behavior. a, Weekly measurements of body weight in grams in basal conditions, FR1 and FR5 (repeated measures ANOVA, genotype effect, **P<0.01). **b,** Body weight (g) of both genotypes in the early and late periods under FR5 (mean ± S.E.M, t-test, **P<0.01). **c-e,** Correlation between the body weight (g) and the three addiction-like criteria **c,** non-reinforced active responses in 10 min, **d,** breaking point in 5h, **e,** number of shocks in 50 min (n=56 for WT mice and n=58 for Glu-CB1-KO mice; statistical details are included in Supplementary Table 1).

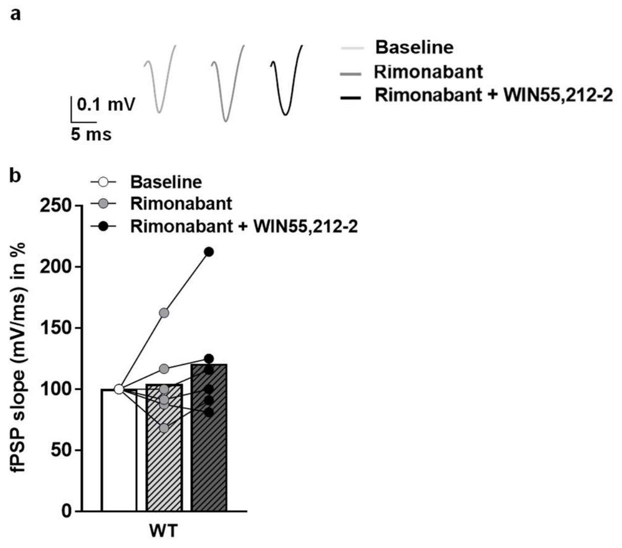

Supplementary Figure 3. Blockade of WIN55,212-2 inhibitory effect in mPFC synaptic transmission by selective CB$_1$R antagonist rimonabant in WT animals. a, Representative recording of field postsynaptic potential (fPSP) in baseline conditions and in the presence of rimonabant (4 µM) and rimonabant (4 µM) + WIN55,212-2 (5 µM) in WT mice (5 slices from 3 mice). **b**, Quantification of the fPSP slope in percentage variation respect to baseline conditions in response to rimonabant (4 µM), and rimonabant (4 µM) + WIN55,212-2 (5 µM). Data was presented as mean and individual values. Statistical details are included in Supplementary Table 2.

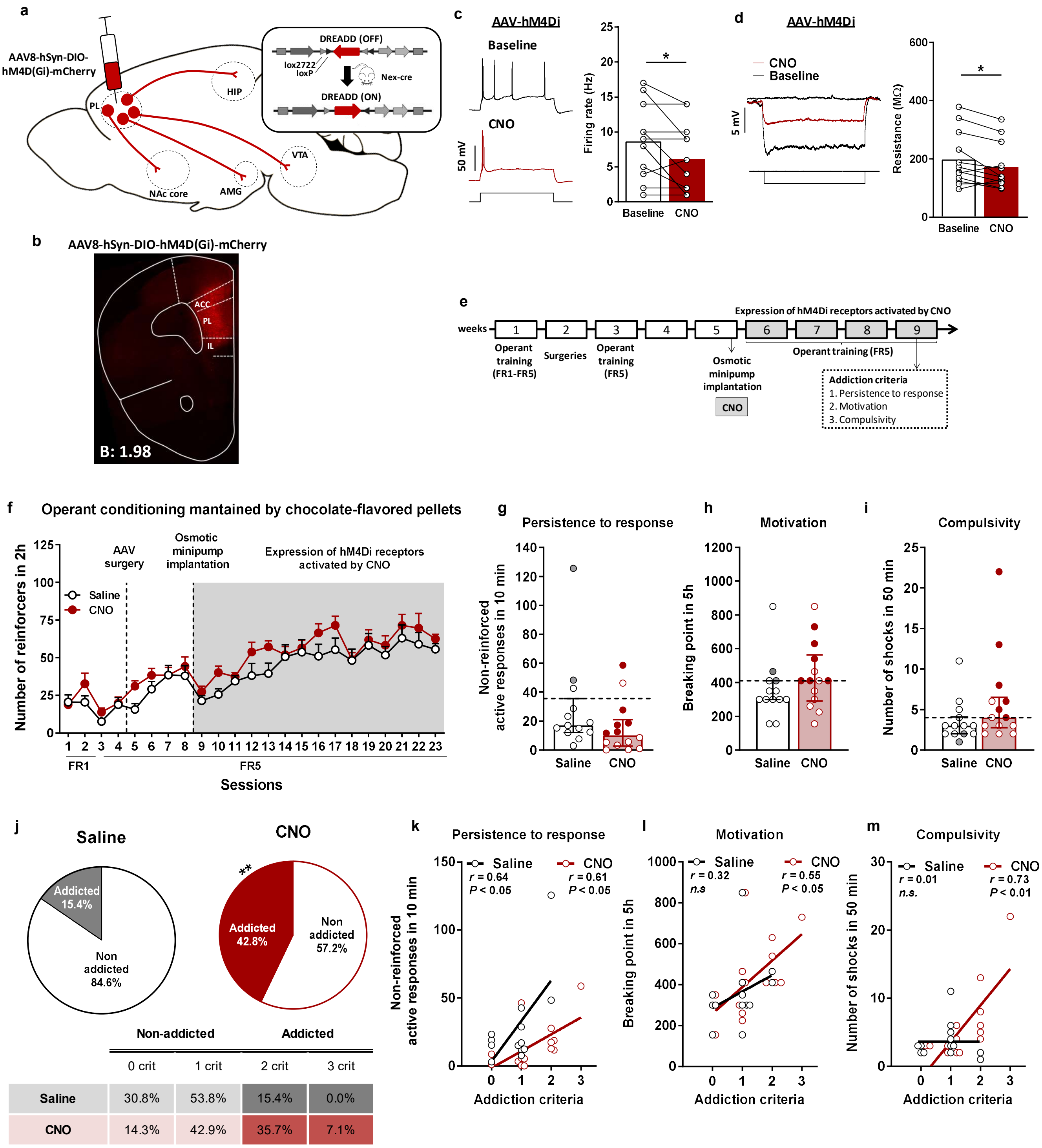

Supplementary Figure 4. Inhibition of glutamatergic PL neurons promotes food addiction-like behavior. a, Scheme of viral strategy for selective hM4Di-mCherry

expression in glutamatergic PL neurons. **b,** Representative immunofluorescence image of Cre-dependent hM4Di-mCherry detected at PL injection site. **c,** Representative recordings showing evoked (200 pA) action potential in visualized hM4Di-mCherry expressing neurons in L5 at baseline and after CNO (10 μM) application (left). Decreased firing rate after CNO application (mean and individual values, paired t-test, *P<0.05; 12 cells from 7 animals; right). **d,** Membrane resistance. Representative recordings showing decreased voltage response to a depolarizing current square pulse of 25 pA (1 s duration) after CNO (10 μM) application compared to baseline in L5 visualized neurons of Nex-Cre mice injected with AAV-hM4Di-mCherry in PL (left). Quantification of the membrane resistance (MΩ) (mean and individual values; Wilcoxon test, *P<0.05; 12 cells from 5 animals; right)**. e,** Timeline of the experimental sequence of the early period of food addiction mouse model. **f,** Number of reinforcers during operant training maintained by chocolate-flavored pellets (mean ± S.E.M). **g-i,** Behavioral tests of the three addiction-like criteria (individual values with the median and the interquartile range). **g**, Persistence to response. **h**, Motivation. **i**, Compulsivity. The 75th percentile of distribution of saline-treated mice is indicated by the dashed horizontal line. Addicted mice in grey filled circles for saline treated mice and red for CNO treated mice. **j,** Increased percentage of CNO treated mice classified as food addicted compared to saline treated animals (chi-square, **P<0.01). **k-m,** Pearson correlations between individual addiction-like criteria and **k,** non-reinforced active responses in 10 min, **l,** breaking point in 5 h, **m,** number of shocks in 50 min (n=13 in saline treated mice and n=14 in CNO treated mice; PL, prelimbic; NAc, nucleus accumbens; Amg, amygdala; Hip, hippocampus; VTA, ventral tegmental area; ACC, anterior cingulate cortex, IL, infralimbic; statistical details are included in Supplementary Table 3).

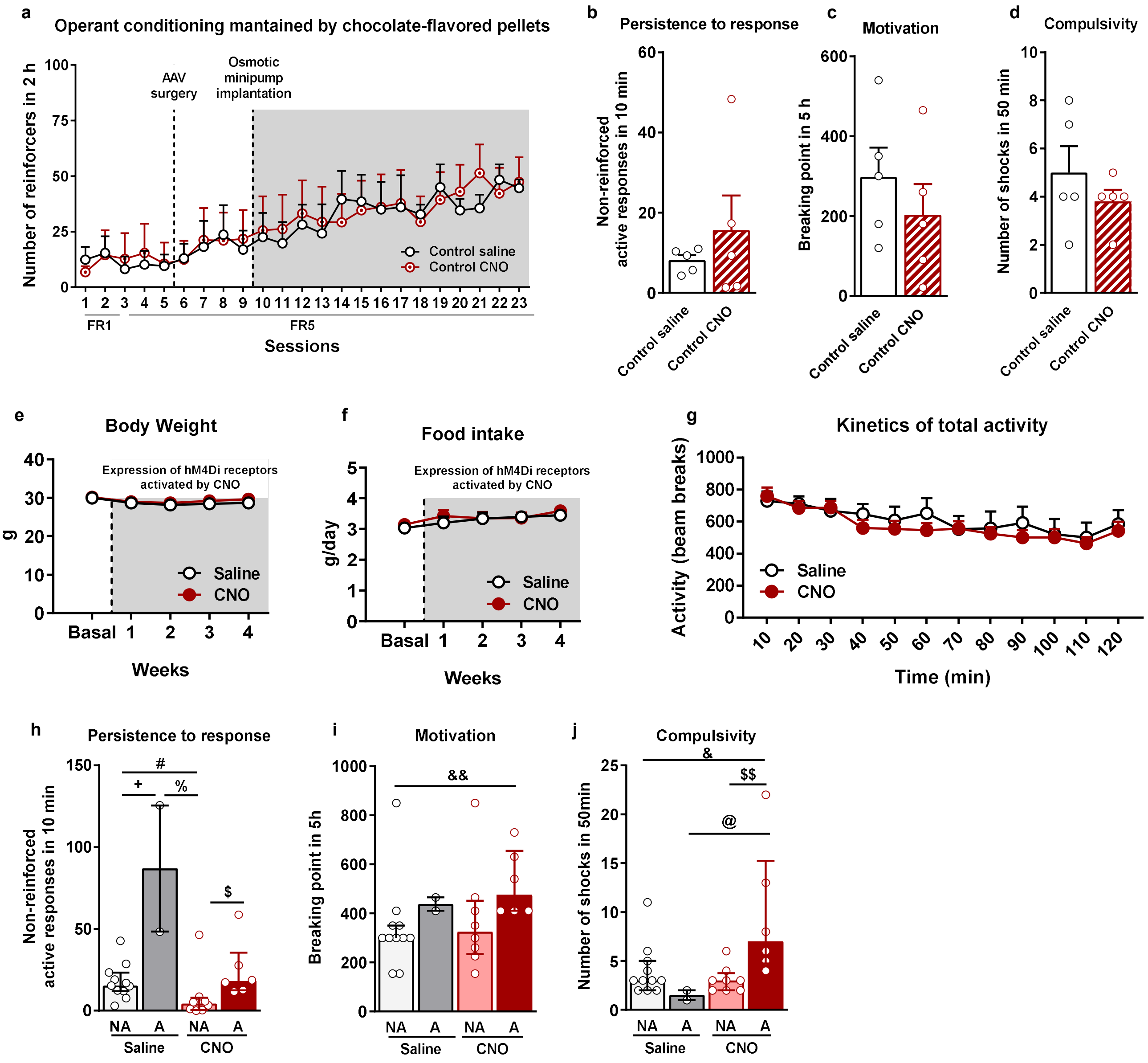

Supplementary Figure 5. CNO treatment do not affect food addiction-like behavior, body weight, food intake nor locomotor activity. **a-d,** Lack of CNO-induced effects in mice injected with AAV-control treated with saline (n=5) or CNO (n=5). **a,** Operant conditioning maintained by chocolate-flavored pellets. **b**, Persistence to response. **c,** Motivation. **d,** Compulsivity. **e-g,** Additional variables to measure the effects of chronically

CNO administration in Nex-Cre mice expressing hM4Di receptors in PL. **e,** Body weight. Weekly measures of body weight in grams for saline and CNO groups. **f,** Food intake. Weekly measures of regular chow food intake provided to mice in their home cage in grams per day for both groups. **g,** Kinetics of total activity. Locomotor activity measured by beam breaks represented in 10-min blocks during 2 h in both groups. **h-j,** Behavioral tests of the three addiction-like criteria represented by individual values and bars with median and the interquartile range for the four groups classified as addicted (A) and non-addicted (NA) mice in both experimental treatment groups. **h,** Persistence to response. **i,** Motivation. **j,** Compulsivity. (U Mann-Whitney, +P< 0.05 saline NA vs saline A; #P< 0.05 saline NA vs CNO NA, &P< 0.05, &&P< 0.01 saline NA vs CNO A, %P< 0.05 saline A vs CNO NA; @P< 0.05 saline A vs CNO A; $P< 0.05, $$P< 0.01 CNO NA vs CNO A; n=13 for saline treated mice and n=14 for CNO treated mice; statistical details are included in Supplementary Table 3).

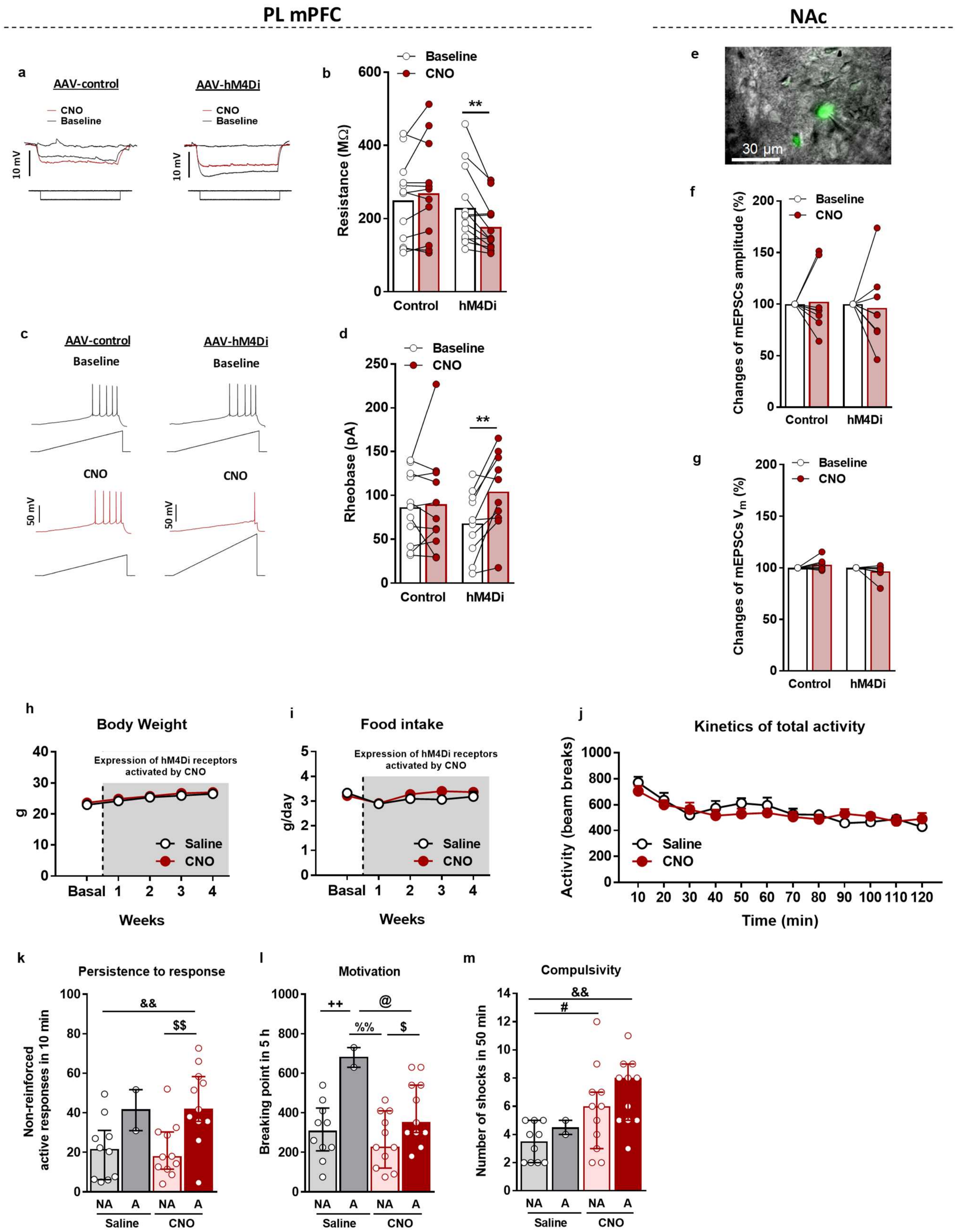
PL mPFC
NAc
a
AAV-control
CNO
Baseline
10 mV
AAV-hM4Di
b
Baseline
CNO
Resistance (MΩ)
Control
hM4Di
c
50 mV
d
Rheobase (pA)
e
30 µm
f
Changes of mEPSCs amplitude (%)
g
Changes of mEPSCs V_m (%)
h
Body Weight
Expression of hM4Di receptors activated by CNO
g
Saline
Basal
Weeks
i
Food intake
g/day
j
Kinetics of total activity
Activity (beam breaks)
Time (min)
k
Persistence to response
Non-reinforced active responses in 10 min
NA
A
l
Motivation
Breaking point in 5 h
m
Compulsivity
Number of shocks in 50 min

Supplementary Figure 6. Inhibition of PL-NAc core pathway leads to compulsivity without affecting body weight, food intake and locomotor activity. a-b, Membrane resistance. **a**, Representative recordings showing decreased voltage response in the PL to a depolarizing current square pulse of 25 pA (1 s duration) after CNO application in mice injected with AAV-control (left) and AAV-hM4Di (right). **b**, Quantification of the membrane resistance (MΩ) (mean with individual values; paired t-test, **P<0.01; 12 cells from 4 mice injected with AAV-control and 14 cells from 4 mice expressing hM4Di)**. c-d,** Rheobase. **c**, Representative recordings showing the increased required current to elicit the first action potential in the PL after CNO application in mice injected with AAV-control (left) and AAV-hM4Di (right). The current ramp was of 150 pA and 1.5 s duration except for mice injected with AAV-hM4Di that was of 300 pA after CNO application. **d**, Quantification of the current required (pA) for firing (mean and individual values,14 cells from 4 mice injected with AAV-control and 10 cells from 3 mice expressing hM4Di; paired t-test, **P<0.01)**. e,** NAc core recorded neuron observed under bright field and green fluorescence conditions**. f-g**, Changes of mEPSCs **f**, amplitude and **g**, resting membrane potential in NAc core in mice injected with AAV-control or AAV-hM4Di in baseline and after CNO application (mean and individual values, 8 cells from 6 mice injected with AAV-control and 8 cells from 7 mice expressing hM4Di). **h-j,** Additional variables to measure the effects of chronic CNO treatment in mice expressing hM4Di receptors in PL-NAc core projection neurons (mean ± S.E.M). **h,** Body weight. Weekly measures of body weight in grams for saline and CNO groups. **i,** Food intake. Weekly measures of regular chow food intake provided to mice in their home cage in grams per day for both groups. **j,** Kinetics of total activity. Locomotor activity measured by beam breaks represented in 10-min blocks during 2 h in both groups.

k-m, Behavioral tests of the three addiction-like criteria represented by individual values and bars with median and the interquartile range for the four groups classified as addicted (A) and non-addicted (NA) mice in both treatment groups. **k,** Persistence to response. **l,** Motivation. **m,** Compulsivity. (t-test and U Mann-Whitney, ++P<0.01 saline NA vs saline A, #P<0.05 saline NA vs CNO NA; &&P<0.01 saline NA vs CNO A, %%P<0.01 saline A vs CNO NA, @P<0.05, $P<0.05, $$P<0.01, CNO NA vs CNO A; n=12 for saline treated mice and n=22 for CNO treated mice; statistical details are included in Supplementary Table 4).

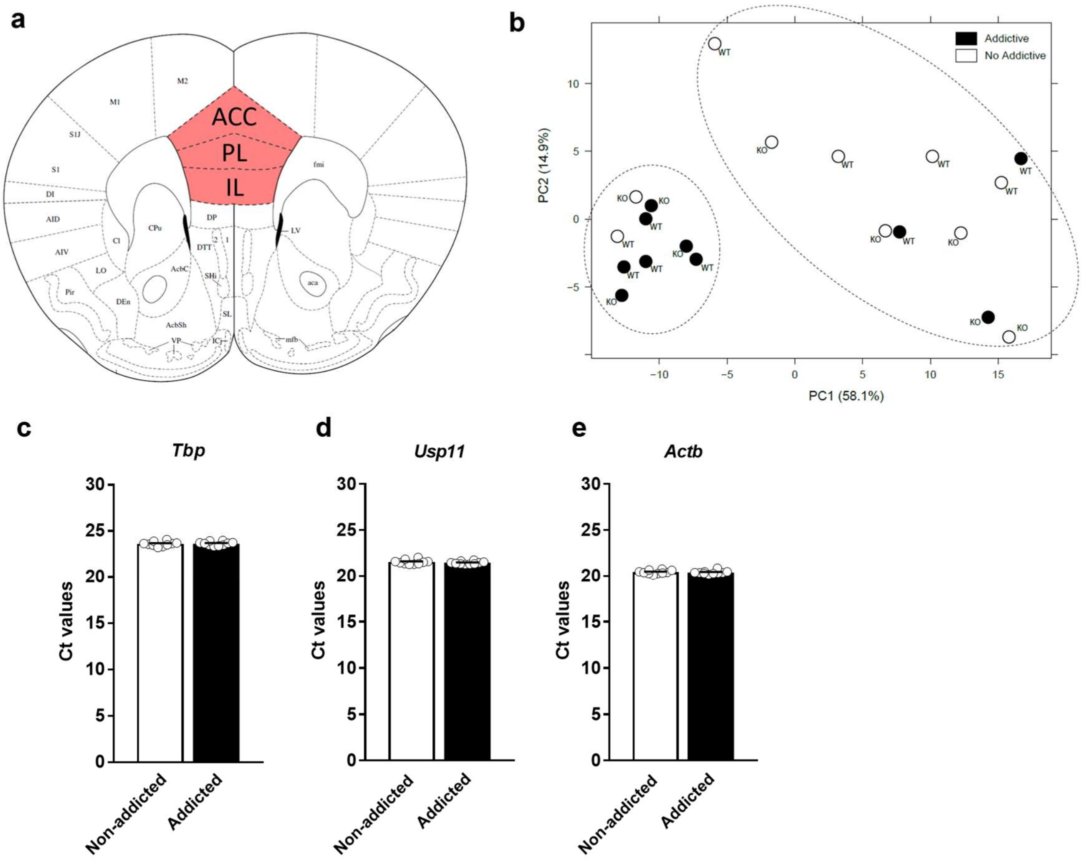

Supplementary Figure 7. a, Schematic diagram of the mPFC area extracted for RNA-seq analysis showing PL: AP +1.98 mm, L ± 0.3 mm, DV -2.3 mm. **b,** Principle component analysis (PCA) showing variation between addicted and non-addicted mice. **c-e,** Ct values for housekeeping genes. ACC, anterior cingulate cortex; PL, prelimbic; IL, infralimbic. Statistical details are included in Supplementary Table 5.

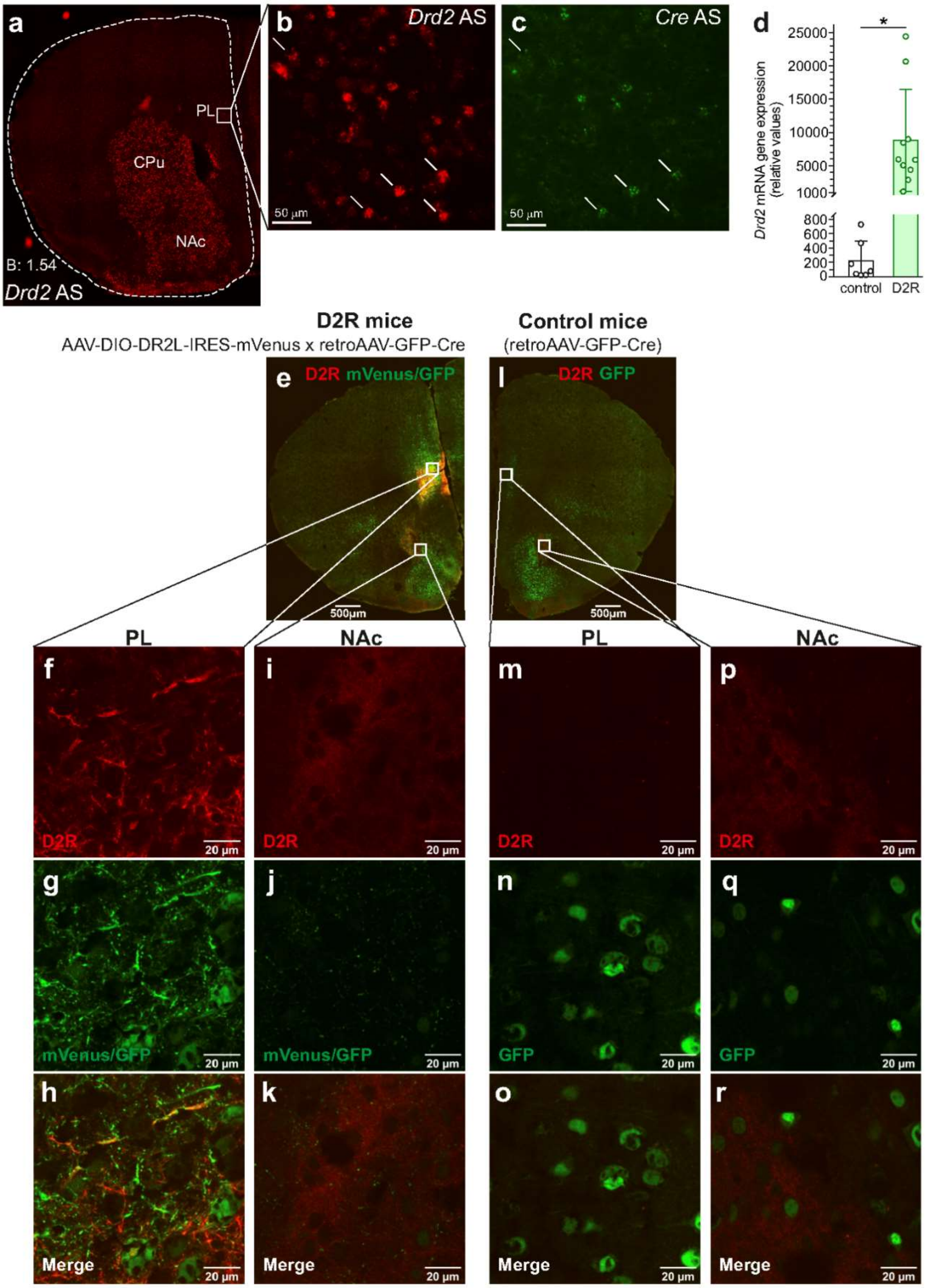
a
PL
CPu
NAc
B: 1.54
Drd2 AS
b
Drd2 AS
50 µm
c
Cre AS
50 µm
d
*
25000
20000
15000
10000
5000
1000
800
600
400
200
0
Drd2 mRNA gene expression (relative values)
control
D2R
D2R mice
AAV-DIO-DR2L-IRES-mVenus x retroAAV-GFP-Cre
Control mice
(retroAAV-GFP-Cre)
e
D2R mVenus/GFP
500µm
l
D2R GFP
500µm
PL
NAc
PL
NAc
f
D2R
i
D2R
m
D2R
p
D2R
g
mVenus/GFP
j
mVenus/GFP
n
GFP
q
GFP
h
Merge
k
Merge
o
Merge
r
Merge
20 µm

Supplementary Figure 8. Colocalization of mRNA *Drd2* and *Cre* recombinase in the PL and protein expression of D_2R, mVenus and Cre in the PL and NAc. a, Overview of *Drd2* mRNA localization (red) in caudate putamen (CPu), NAc and PL at bregma of approximately: 1.54 mm, as detected by *in situ* hybridization. **b,** Enlarged view of area shown in panel **a**, revealing *Drd2* mRNA localization (red) in PL, and **c,** *Cre* mRNA (green). Arrows: Cells with colocalization of *Drd2* and *Cre* mRNA; arrowheads: Cells expressing only *Drd2* mRNA. Quantification of cells co-expressing Drd2 and Cre mRNA (n=2). About 50% of Drd2 mRNA expressing cells revealed co-expression with Cre mRNA. **d**, Overexpression of *Drd2* gene in PL-NAc projections. Quantitative real time PCR in mPFC of *Drd2* mRNA levels in control (n=7) and D_2R-overexpressing mice (n=10). **e-r**, Imunohistological analysis of brain sections from mice injected with AAVrg-Cre-GFP into NAc core and with AAV-D_2R-mVenus into PL, detecting D_2R (red) and mVenus/GFP (green). **e,** Overview of D_2R, mVenus and GFP distribution in the forebrain at the level of mPFC in D_2R overexpressing mice; **f-h**, enlarged areas from PL as indicated in panel e, showing overexpressed D_2R in the neuropil of PL neurons at dendritic postsynaptic site. **i-k**, enlarged areas from NAc as indicated in panel e, showing mVenus/GFP expression in NAc at axonal terminal sites of PL-NAc projections. **l,** Overview of D_2R and GFP distribution in the forebrain at the level of mPFC in control mice; **m-o**, enlarged areas from PL as indicated in in panel l, showing no D_2R overexpression in PL neurons. **p-r**, enlarged areas from NAc as indicated in panel l, showing GFP-Cre expression in NAc and the lack of overexpressed D_2R. Scale bars: 50 µm in b and c, 500 µm in e and l, and 20 µm in f-k and m-r. Statistical details are included in Supplementary Table 6.

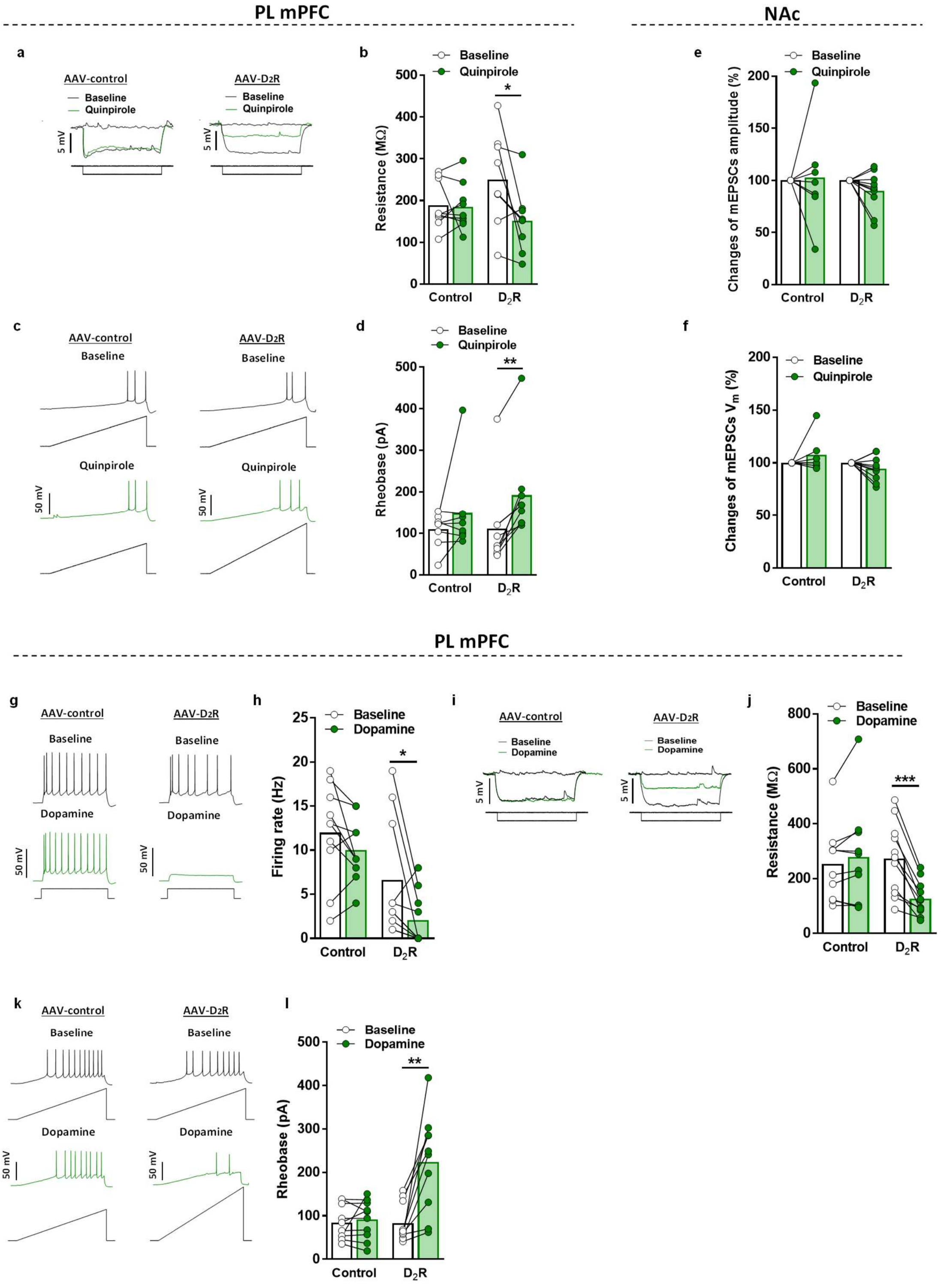
PL mPFC
NAc
a
AAV-control
Baseline
Quinpirole
5 mV
AAV-D2R
Baseline
Quinpirole
5 mV
b
Baseline
Quinpirole
Resistance (MΩ)
*
Control
D2R
e
Baseline
Quinpirole
Changes of mEPSCs amplitude (%)
Control
D2R
c
AAV-control
Baseline
Quinpirole
50 mV
AAV-D2R
Baseline
Quinpirole
50 mV
d
Baseline
Quinpirole
Rheobase (pA)
**
Control
D2R
f
Baseline
Quinpirole
Changes of mEPSCs Vm (%)
Control
D2R
PL mPFC
g
AAV-control
Baseline
Dopamine
50 mV
AAV-D2R
Baseline
Dopamine
50 mV
h
Baseline
Dopamine
Firing rate (Hz)
*
Control
D2R
i
AAV-control
Baseline
Dopamine
5 mV
AAV-D2R
Baseline
Dopamine
5 mV
j
Baseline
Dopamine
Resistance (MΩ)
***
Control
D2R
k
AAV-control
Baseline
Dopamine
50 mV
AAV-D2R
Baseline
Dopamine
50 mV
l
Baseline
Dopamine
Rheobase (pA)
**
Control
D2R

Supplementary Figure 9. Electrophysiological recordings confirm *Drd2* overexpression in PL-NAc core pathway. Electrophysiological recordings from WT mice injected with AAV-control and overexpressing D_2R (injected with AAV-D_2R) in PL L5 visualized neurons and NAc core at baseline and after quinpirole (2 μM) or dopamine (10 μM) application represented as mean and individual data. **a-b,** Membrane resistance. **a**, Representative recordings showing decreased voltage response to a depolarizing current square pulse of 25 pA (1 s duration) after quinpirole application in mice injected with AAV-control (left) and AAV- D_2R (right). **b**, Quantification of the membrane resistance (MΩ) (9 cells from 3 mice injected with AAV-control and 9 cells from 3 mice overexpressing D_2R; paired t-test, $^*P<0.05$)**. c-d,** Rheobase. **c**, Representative recordings showing the increased required current to elicit the first action potential after quinpirole application in mice injected with AAV-control (left) and AAV- D_2R (right). The current ramp was of 150 pA and 1.5 s duration except for mice injected with AAV-D_2R that was of 250 pA after quinpirole application. **d**, Quantification of the current required (pA) for firing (8 cells from 3 mice injected with AAV-control and 9 cells from 3 mice overexpressing D_2R; Wilcoxon test, $^{**}P<0.01$)**. e-f**, Changes of mEPSCs **e**, amplitude and **f**, resting membrane in the NAc core in mice injected with AAV-control or AAV-D_2R at baseline and after quinpirole application (7 cells from 5 mice injected with AAV-control and 10 cells from 5 mice overexpressing D_2R)**. g-h,** Firing rate. **g,** Representative recordings showing evoked (150 pA) action potential after dopamine application in mice injected with AAV-control (left) and AAV- D_2R (right). **h,** Quantification of the firing rate (Hz) (Wilcoxon, $^*P<0.05$; 10 cells from 3 mice injected with AAV-control and 10 cells from 3 mice overexpressing D_2R). **i-j,** Membrane resistance. **i**, Representative recordings showing decreased voltage response to a depolarizing current square pulse of 25

pA (1 s duration) after dopamine application in mice injected with AAV-control (left) and AAV- D_2R (right). **j,** Quantification of the membrane resistance (MΩ) (paired t-test, ***P<0.001; 10 cells from 3 mice injected with AAV-control and 11 cells from 3 mice overexpressing D_2R;)**. k-l,** Rheobase. **k**, Representative recordings showing the increased required current to elicit the first action potential after dopamine application in mice injected with AAV-control (left) and AAV- D_2R (right). The current ramp was of 150 pA and 1.5 s duration except for mice injected with AAV-D_2R that was of 250 pA after dopamine application. **l,** Quantification of the current required (pA) for firing (right, Wilcoxon test, **P<0.01; 10 cells from 3 mice injected with AAV-control and 10 cells from 3 mice overexpressing D_2R; Wilcoxon test)**.** Statistical details are included in Supplementary Table 6.

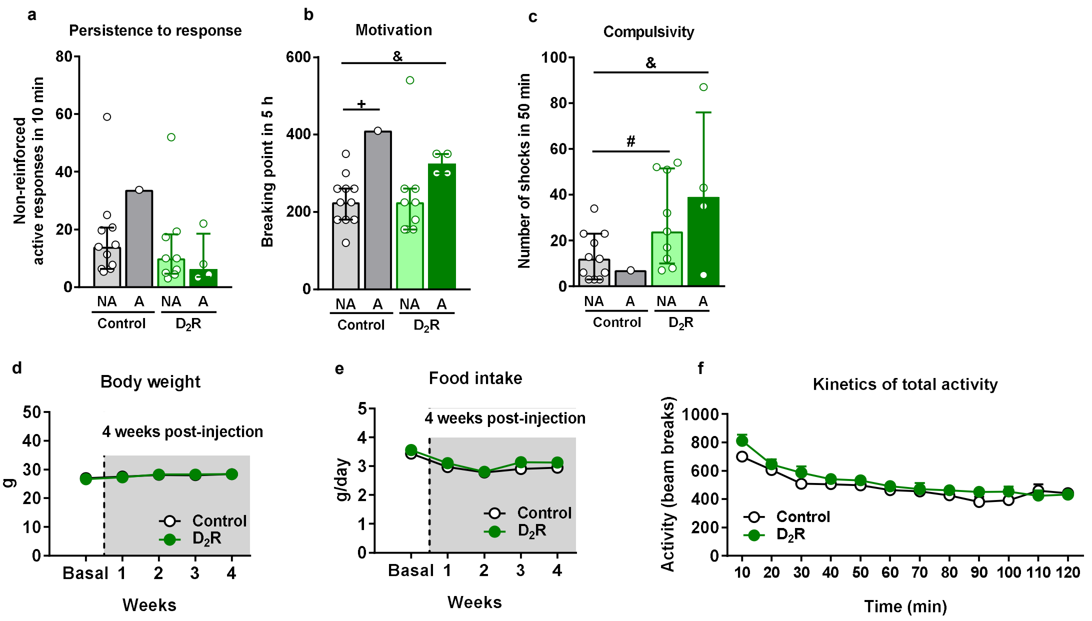

Supplementary Figure 10. *Drd2* overexpression in PL-NAc core pathway promotes compulsivity without affecting body weight, food intake and locomotor activity. a-c, Behavioral tests of the three addiction-like criteria represented by individual values and bars with median and the interquartile range for the four groups classified as addicted (A) and non-addicted (NA) mice in both injected groups. **a,** Persistence to response. **b,** Motivation. **c,** Compulsivity (U Mann-Whitney, +P<0.05 control NA vs control A, #P<0.05 control NA vs D_2R NA, &P<0.05 control NA vs D_2R A). **d-f,** Control variables to measure the effects of D_2R overexpression in mice overexpressing D_2R in PL-NAc core projection neurons. **d,** Body weight. Weekly measures of body weight in grams for both injected groups. **e,** Food intake. Weekly measures of regular chow food intake provided to mice in their home cage in grams per day for both groups. **f,** Kinetics of total activity. Locomotor activity measured by beam breaks represented in 10-min blocks during 2 hours in both injected groups (n=12 for

mice injected with AAV-control mice and n=13 for mice injected with AAV-D_2R mice; statistical details are included in Supplementary Table 6).

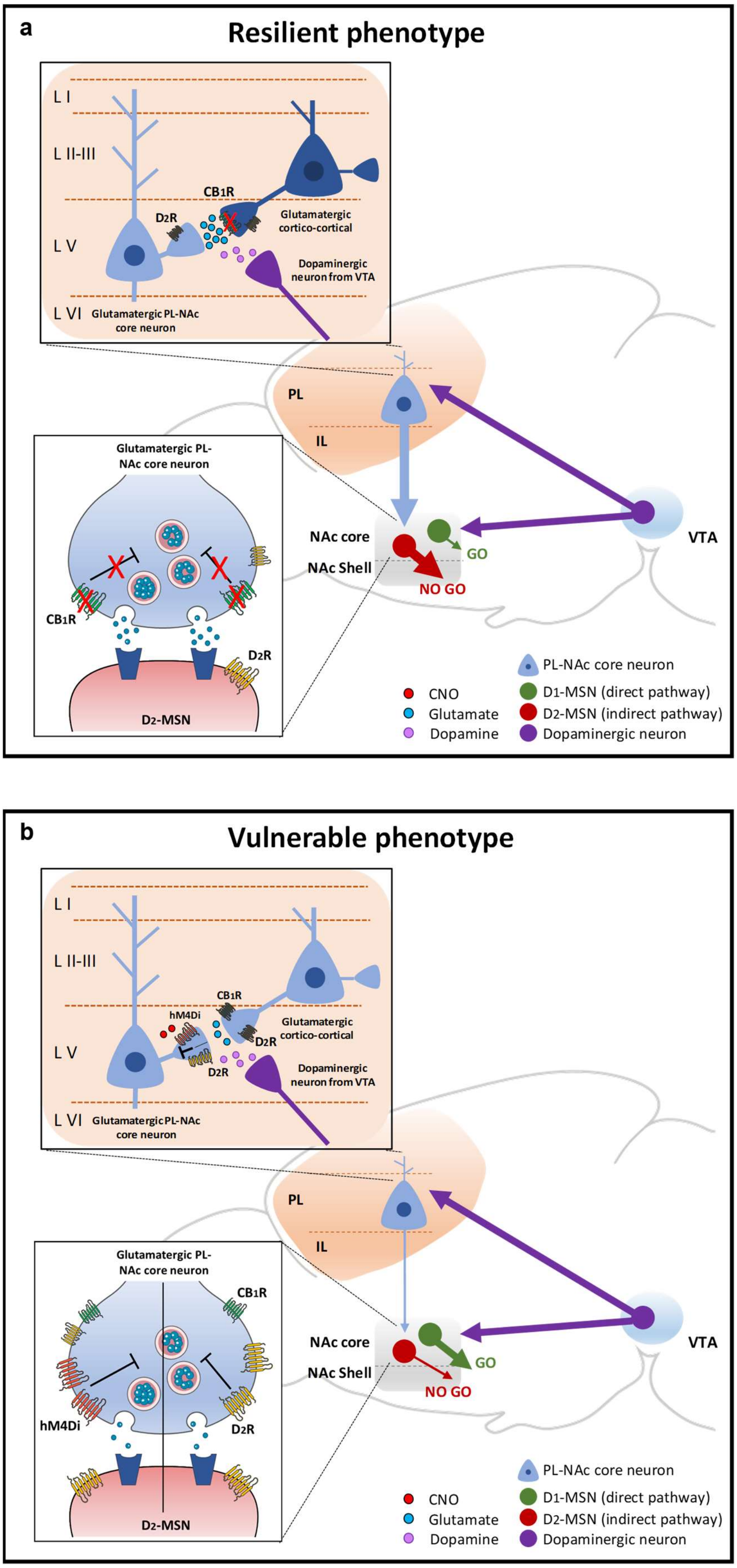

a
Resilient phenotype
L I
L II-III
L V
L VI
CB1R
D2R
Glutamatergic cortico-cortical
Dopaminergic neuron from VTA
Glutamatergic PL-NAc core neuron
PL
IL
Glutamatergic PL-NAc core neuron
CB1R
D2R
D2-MSN
NAc core
NAc Shell
GO
NO GO
VTA
CNO
Glutamate
Dopamine
PL-NAc core neuron
D1-MSN (direct pathway)
D2-MSN (indirect pathway)
Dopaminergic neuron
b
Vulnerable phenotype
L I
L II-III
L V
L VI
CB1R
hM4Di
D2R
D2R
Glutamatergic cortico-cortical
Dopaminergic neuron from VTA
Glutamatergic PL-NAc core neuron
PL
IL
Glutamatergic PL-NAc core neuron
CB1R
hM4Di
D2R
D2-MSN
NAc core
NAc Shell
GO
NO GO
VTA
CNO
Glutamate
Dopamine
PL-NAc core neuron
D1-MSN (direct pathway)
D2-MSN (indirect pathway)
Dopaminergic neuron

Supplementary Figure 11. Schematic summary of the PL-NAc core pathway regulation of resilience and vulnerability to develop food addiction. a, Resilient phenotype. Deletion of the CB_1R in dorsal telencephalic glutamatergic neurons increased glutamate release in local cortical networks increasing excitatory glutamatergic transmission in L5 prelimbic neurons projecting to NAc core. Subsequently, the increased glutamatergic transmission from cortical pyramidal neurons could stimulated D2-MSN indirect pathway (NO GO response) in NAc core facilitating the avoidance behavior. **b,** Vulnerable phenotype. Overexpression of hM4Di receptors or D_2Rs in PL neurons projecting to NAc core and the subsequent activation of these receptors by CNO and dopamine, respectively, produced a decreased excitatory transmission of this network, thereby possibly reducing the activation of D2-MSN indirect pathway in NAc core. The decreased activity of the indirect pathway suppressed the avoidance behavior (NO GO response) facilitating the D1-MSN direct pathway activity promoting the approach behavior (GO response). PL, prelimbic; IL, infralimbic; NAc, nucleus accumbens; VTA, ventral tegmental area; D1-MSN, dopaminergic D1 medium spiny neuron; D2-MSN, dopamine D2 medium spiny neuron; D_2R, dopamine D2 receptor; hM4Di, human muscarinic 4 designer inhibitory G_i receptor; CB_1R, cannabinoid type-1 receptor.

Supplementary Tables

Supplementary Table 1. Statistical details of experiments shown in Fig. 1 and Supplementary Figure 1 and 2

Glu-CB1-KO mice display resilience to food addiction				
**Figure number**	**Statistical analysis**	**Factor name**	**Statistic value**	**P-value**
Fig. 1b	Repeated measures ANOVA	**FR1 (Sessions 1-6)**		
		Genotype	$F_{(1,112)} = 0.33$	n.s
		Sessions	$F_{(5,560)} = 29.00$	$P < 0.001$
		Genotype x Sessions	$F_{(5,560)} = 0.62$	n.s
		**FR5 (Sessions 1-112)**		
		Genotype	$F_{(1,112)} = 36.72$	$P < 0.001$
		Sessions	$F_{(111,12432)} = 5.38$	$P < 0.001$
		Genotype x Sessions	$F_{(111,12432)} = 3.53$	$P < 0.001$
Fig. 1c-e	Kolmogorov-Smirnov	**Late period**		
		Persistence to response	$K\text{-}S = 0.25$	$P < 0.001$
		Motivation	$K\text{-}S = 0.16$	$P < 0.001$
		Compulsivity	$K\text{-}S = 0.28$	$P < 0.001$
	U Mann-Whitney	**Late period**		
		Persistence to response	$U = 1043.50$	$P < 0.01$
		Motivation	$U = 1035.50$	$P < 0.01$
		Compulsivity	$U = 1071.00$	$P < 0.01$
Fig. 1f	Chi-square	Genotype	$C\text{-}S = 7.06$	$P < 0.01$
Fig. 1g-i	Pearson correlation	**WT**		
		Non-reinforced active responses in 10 min and addiction criteria	$r = 0.74$	$P < 0.001$
		Breaking point in 5h and addiction criteria	$r = 0.73$	$P < 0.001$
		Compulsivity and addiction criteria	$r = 0.46$	$P < 0.001$
		**Glu-CB1-KO**		
		Non-reinforced active responses in 10 min and addiction criteria	$r = 0.53$	$P < 0.001$
		Breaking point in 5h and addiction criteria	$r = 0.73$	$P < 0.001$
		Compulsivity and addiction criteria	$r = 0.46$	$P < 0.001$
Supplementary Fig. 1a	Kolmogorov-Smirnov	**Pellets intake**		
		FR1	$K\text{-}S = 0.10$	$P < 0.05$
		FR5 Early period	$K\text{-}S = 0.08$	n.s.
		FR5 Late period	$K\text{-}S = 0.05$	n.s.
		**Pellets intake**		
	U Mann-Whitney	FR1	$U = 1545.5$	n.s.
	t-test (Equal variances assumed)	FR5 Early period	$t = 3.06$	$P < 0.01$
		FR5 Late period	$t = 6.58$	$P < 0.001$
Supplementary Fig. 1b-d	Kolmogorov-Smirnov	**Early period**		
		Persistence to response	$K\text{-}S = 0.15$	$P < 0.001$
		Motivation	$K\text{-}S = 0.19$	$P < 0.001$
		Compulsivity	$K\text{-}S = 0.27$	$P < 0.001$
	U Mann-Whitney	**Early period**		
		Persistence to response	$U = 1320.00$	n.s.
		Motivation	$U = 1384.50$	n.s.
		Compulsivity	$U = 1242.50$	$P < 0.05$

Supplementary Fig. 1e	Kolmogorov-Smirnov	**Early period**		
		Impulsivity	*K-S* = 0.22	*P* < 0.001
		**Late period**		
		Impulsivity	*K-S* = 0.21	*P* < 0.001
	U Mann-Whitney	**Early period**		
		Impulsivity	*U* = 1464.00	n.s
		**Late period**		
		Impulsivity	*U* = 1159.50	*P* < 0.01
Supplementary Fig. 1f	Kolmogorov-Smirnov	**Early period**		
		Shock-induced suppression	*K-S* = 0.14	*P* < 0.001
		**Late period**		
		Shock-induced suppression	*K-S* = 0.20	*P* < 0.001
	U Mann-Whitney	**Early period**		
		Shock-induced suppression	*U* = 1178.50	*P* < 0.05
		**Late period**		
		Shock-induced suppression	*U* = 697.00	*P* < 0.001
Supplementary Fig. 1g-k	Kolmogorov-Smirnov	Persistence to response	*K-S* = 0.25	*P* < 0.001
		Motivation	*K-S* = 0.16	*P* < 0.001
		Compulsivity	*K-S* = 0.28	*P* < 0.001
		Impulsivity	*K-S* = 0.21	*P* < 0.001
		Shock-induced suppression	*K-S* = 0.21	*P* < 0.001
	U Mann-Whitney	**Persistence to response**		
		WT NA vs WT A	*U* = 40.50	*P* < 0.001
		WT NA vs Glu-CB1-KO NA	*U* = 866.50	*P* < 0.05
		WT NA vs Glu-CB1-KO A	*U* = 52.00	n.s.
		WT A vs Glu-CB1-KO NA	*U* = 42. 00	*P* < 0.001
		WT A vs Glu-CB1-KO A	*U* = 19.00	n.s.
		Glu-CB1-KO NA vs Glu-CB1-KO A	*U* = 57.50	n.s.
		**Motivation**		
		WT NA vs WT A	*U* = 52.50	*P* < 0.001
		WT NA vs Glu-CB1-KO NA	*U* = 803.50	*P* < 0.05
		WT NA vs Glu-CB1-KO A	*U* = 10.00	*P* < 0.01
		WT A vs Glu-CB1-KO NA	*U* = 52.50	*P* < 0.001
		WT A vs Glu-CB1-KO A	*U* = 18.50	n.s.
		Glu-CB1-KO NA vs Glu-CB1-KO A	*U* = 14.50	*P* < 0.01
		**Compulsivity**		
		WT NA vs WT A	*U* = 80.00	*P* < 0.001
		WT NA vs Glu-CB1-KO NA	*U* = 814.50	*P* < 0.05
		WT NA vs Glu-CB1-KO A	*U* = 7.50	*P* < 0.01
		WT A vs Glu-CB1-KO NA	*U* = 61.50	*P* < 0.001
		WT A vs Glu-CB1-KO A	*U* = 21.50	n.s.
		Glu-CB1-KO NA vs Glu-CB1-KO A	U = 4.00	*P* < 0.01
		**Impulsivity**		
		WT NA vs WT A	*U* = 102.00	*P* < 0.001
		WT NA vs Glu-CB1-KO NA	*U* = 878.50	n.s.
		WT NA vs Glu-CB1-KO A	*U* = 33.00	*P* < 0.05
		WT A vs Glu-CB1-KO NA	*U* = 107.00	*P* < 0.001
		WT A vs Glu-CB1-KO A	*U* = 17.00	n.s.
		Glu-CB1-KO NA vs Glu-CB1-KO A	*U* = 37.00	*P* < 0.05

Supplementary Fig. 1g-k	U Mann-Whitney	**Shock-induced suppression**<br>WT NA vs WT A	$U = 185.50$	$P < 0.05$
		WT NA vs Glu-CB1-KO NA	U = 439.50	$P < 0.001$
		WT NA vs Glu-CB1-KO A	$U = 61.50$	n.s.
		WT A vs Glu-CB1-KO NA	$U = 125.00$	$P < 0.001$
		WT A vs Glu-CB1-KO A	$U = 26.00$	n.s.
		Glu-CB1-KO NA vs Glu-CB1-KO A	$U = 36.50$	$P < 0.05$
Supplementary Fig. 2a	Repeated measures ANOVA	**Body weight**		
		Genotype	$F_{(1,112)} = 7.02$	$P < 0.01$
		Sessions	$F_{(23,2576)} = 231.81$	$P < 0.001$
		Genotype x Sessions	$F_{(23,2576)} = 4.44$	$P < 0.001$
Supplementary Fig. 2b	Kolmogorov-Smirnov	Early period	$K\text{-}S = 0.07$	n.s.
		Late period	$K\text{-}S = 0.06$	n.s.
	t-test	Early period	$t = 4.23$	n.s
		Late period	$t = 0.19$	$P < 0.01$
Supplementary Fig. 2c-e	Pearson correlation	**WT**		
		Non-reinforced active responses in 10 min and addiction criteria	$r = -0.16$	n.s
		Breaking point in 5h and addiction criteria	$r = 0.06$	n.s
		Compulsivity and addiction criteria	$r = -0.04$	n.s
		**Glu-CB1-KO**		
		Non-reinforced active responses in 10 min and addiction criteria	$r = -0.11$	n.s
		Breaking point in 5h and addiction criteria	$r = -0.14$	n.s
		Compulsivity and addiction criteria	$r = -0.14$	n.s

Supplementary Table 2. Statistical details of experiments shown in Fig. 2 and Supplementary Figure 3

Synaptic excitatory transmission is increased in Glu-CB1-KO				
**Figure number**	**Statistical analysis**	**Factor name**	**Statistic value**	**P-value**
Fig. 2c-d and f-g	Kolmogorov-Smirnov	**PL mPFC**		
		mEPSC frequency	*K-S* = 0.14	n.s.
		mEPSC amplitude	*K-S* = 0.16	n.s.
		mIPSC frequency	*K-S* = 0.17	n.s.
		mIPSC amplitude	*K-S* = 0.16	n.s.
	t-test (Equal variances assumed)	**PL mPFC**		
		mEPSC frequency	t = -4.09	$P < 0.01$
		mEPSC amplitude	t = 1.77	n.s.
		mIPSC frequency	t = 0.13	n.s.
		mIPSC amplitude	t = 0.09	n.s.
Fig. 2j-k and m-n	Kolmogorov-Smirnov	**NAc**		
		mEPSC frequency	*K-S* = 0.12	n.s.
		mEPSC amplitude	*K-S* = 0.17	n.s.
		mIPSC frequency	*K-S* = 0.17	n.s.
		mIPSC amplitude	*K-S* = 0.13	n.s.
	t-test (Equal variances assumed)	**NAc**		
		mEPSC frequency	t = -2.46	$P < 0.05$
		mEPSC amplitude	t = 0.73	n.s.
		mIPSC frequency	t = -0.15	n.s.
		mIPSC amplitude	t = 1.23	n.s.
Fig. 2p	Kolmogorov-Smirnov	PPF	*K-S* = 0.13	n.s.
	U Mann-Whitney	PPF	t = -3.28	$P < 0.01$
Fig. 2r	Kolmogorov-Smirnov	WIN55,212-2	*K-S* = 0.16	n.s.
	Paired t-test	**fPSP amplitude (%)**		
		WT	t = 8.38	$P < 0.001$
		Glu-CB1-KO	t = -1.75	n.s.
		WT vs Glu-CB1-KO	t = -7.28	$P < 0.001$
Fig. 2t	Kolmogorov-Smirnov	WIN55,212-2	*K-S* = 0.13	n.s.
	Paired t-test	**EPSCs amplitude (%)**		
		WT	t = 6.35	$P < 0.001$
		Glu-CB1-KO	t = 4.04	$P < 0.01$
		WT vs Glu-CB1-KO	t = -3.36	$P < 0.01$
Supplementary Fig. 3b	Kolmogorov-Smirnov	**fPSP slope (%)**		
		Baseline	*K-S* = 0.25	n.s
		Rimonabant	*K-S* = 0.26	n.s
		Rimonabant + WIN	*K-S* = 0.20	n.s
	Friedman test	fPSP slope (%)	*C-S* = 2.27	n.s

Supplementary Table 3. Statistical details of experiments shown in Supplementary Figure 4 and 5

Inhibition of glutamatergic PL neurons promotes food addiction-like behavior				
**Figure number**	**Statistical analysis**	**Factor name**	**Statistic value**	**P-value**
Supplementary Fig. 4c		**AAV-hM4Di**		
	Kolmogorov-Smirnov	Firing rate Baseline	*K-S* = 0.15	n.s.
		Firing rate CNO	*K-S* = 0.21	n.s.
	Paired t-test	Firing rate	$t = 2.66$	$P < 0.05$
Supplementary Fig. 4d	Kolmogorov-Smirnov	Resistance baseline	*K-S* = 0.21	n.s.
		Resistance CNO	*K-S* = 0.25	$P < 0.05$
	Wilcoxon test	Resistance	$Z = -2.12$	$P < 0.05$
Supplementary Fig. 4f	Repeated measures ANOVA	**FR1 (Sessions 1-2)**		
		Treatment	$F_{(1,25)} = 0.70$	n.s
		Sessions	$F_{(1,25)} = 4.01$	n.s
		Treatment x Sessions	$F_{(1,25)} = 4.16$	n.s
		**FR5 (Sessions 3-8)**		
		Treatment	$F_{(1,25)} = 1.34$	n.s
		Sessions	$F_{(5,125)} = 29.85$	$P < 0.001$
		Treatment x Sessions	$F_{(5,125)} = 1.69$	n.s
		**FR5 (Sessions 9-23)**		
		Treatment	$F_{(1,24)} = 2.16$	n.s
		Sessions	$F_{(14,350)} = 12.82$	$P < 0.001$
		Treatment x Sessions	$F_{(14,350)} = 0.75$	n.s
Supplementary Fig. 4g-i	Kolmogorov-Smirnov	Persistence to response	*K-S* = 0.25	$P < 0.001$
		Motivation	*K-S* = 0.22	$P < 0.01$
		Compulsivity	*K-S* = 0.25	$P < 0.001$
	U Mann-Whitney	Persistence to response	$U = 57.00$	*n.s*
		Motivation	$U = 64.50$	*n.s.*
		Compulsivity	$U = 61.50$	*n.s.*
Supplementary Fig. 4j	Chi square	Treatment	*C-S* = 8.12	$P < 0.01$
Supplementary Fig. 4k-m	Pearson correlation	**Saline**		
		Non-reinforced active responses in 10 min and addiction criteria	$r = 0.64$	$P < 0.05$
		Breaking point in 5h and addiction criteria	$r = 0.32$	n.s.
		Compulsivity and addiction criteria	$r = 0.01$	n.s.
		**CNO**		
		Non-reinforced active responses in 10 min and addiction criteria	$r = 0.61$	$P < 0.05$
		Breaking point in 5h and addiction criteria	$r = 0.56$	$P < 0.05$
		Compulsivity and addiction criteria	$r = 0.74$	$P < 0.01$
Supplementary Fig. 5a	Repeated measures ANOVA	**FR1 (Sessions 1-2)**		
		Treatment	$F_{(1,8)} = 0.14$	n.s
		Sessions	$F_{(8,64)} = 0.87$	n.s
		Treatment x Sessions	$F_{(8,64)} = 0.16$	n.s
		**FR5 (Sessions 6-9)**		
		Treatment	$F_{(1,8)} = 0.03$	n.s
		Sessions	$F_{(8,64)} = 2.45$	$P < 0.05$
		Treatment x Sessions	$F_{(8,64)} = 0.23$	n.s

Supplementary Fig. 5a	Repeated measures ANOVA	**FR5 (Sessions 10-23)**		
		Treatment	$F_{(1,8)} = 0.01$	n.s
		Sessions	$F_{(13,104)} = 2.15$	$P < 0.05$
		Treatment x Sessions	$F_{(13,104)} = 0.45$	n.s
Supplementary Fig. 5b-d	Kolmogorov-Smirnov	**Addiction criteria**		
		Persistence to response	$K\text{-}S = 0.33$	$P < 0.01$
		Motivation	$K\text{-}S = 0.16$	n.s.
		Compulsivity	$K\text{-}S = 0.28$	$P < 0.05$
	U Mann-Whitney	**Addiction criteria**		
		Persistence to response	$U = 12.50$	n.s.
	t-test (Equal variances assumed)	Motivation	$t = 0.89$	n.s.
	U Mann-Whitney	Compulsivity	$U = 9.50$	n.s.
Supplementary Fig. 5e-g	Repeated measures ANOVA	**Body Weight**		
		Treatment	$F_{(1,27)} = 0.61$	n.s
		Weeks	$F_{(4,108)} = 7.46$	$P < 0.001$
		Treatment x Weeks	$F_{(4,108)} = 0.44$	n.s
		**Food Intake**		
		Treatment	$F_{(1,27)} = 0.16$	n.s
		Weeks	$F_{(3,81)} = 1.08$	n.s
		Treatment x Weeks	$F_{(3,81)} = 0.45$	n.s
		**Kinetics of total activity**		
		Treatment	$F_{(1,16)} = 0.21$	n.s
		Time	$F_{(11,176)} = 11.20$	$P < 0.001$
		Treatment x Time	$F_{(11,176)} = 0.84$	n.s
Supplementary Fig. 5h-j	Kolmogorov-Smirnov	Persistence to response	$K\text{-}S = 0.71$	$P < 0.001$
		Motivation	$K\text{-}S = 0.88$	$P < 0.01$
		Compulsivity	$K\text{-}S = 0.68$	$P < 0.001$
	U Mann-Whitney	**Persistence to response**		
		Saline NA vs Saline A	$U = 00.00$	$P < 0.05$
		Saline NA vs CNO NA	$U = 16.00$	$P < 0.05$
		Saline NA vs CNO A	$U = 26.00$	n.s.
		Saline A vs CNO NA	$U = 0.00$	$P < 0.05$
		Saline A vs CNO A	$U = 1.00$	n.s.
		CNO NA vs CNO A	$U = 5.00$	$P < 0.05$
		**Motivation**		
		Saline NA vs Saline A	$U = 2.50$	n.s.
		Saline NA vs CNO NA	$U = 40.50$	n.s.
		Saline NA vs CNO A	$U = 7.50$	$P < 0.01$
		Saline A vs CNO NA	$U = 4.00$	n.s.
		Saline A vs CNO A	$U = 4.50$	n.s.
		CNO NA vs CNO A	$U = 10.50$	n.s.
		**Compulsivity**		
		Saline NA vs Saline A	$U = 1.50$	n.s.
		Saline NA vs CNO NA	$U = 36.50$	n.s.
		Saline NA vs CNO A	$U = 8.50$	$P < 0.05$
		Saline A vs CNO NA	$U = 1.50$	n.s.
		Saline A vs CNO A	$U = 0.00$	$P < 0.05$
		CNO NA vs CNO A	$U = 3.00$	$P < 0.01$

Supplementary Table 4. Statistical details of experiments shown in Fig. 3 and Supplementary Figure 6

Inhibition of PL-NAc core projection pathway leads to compulsivity				
**Figure number**	**Statistical analysis**	**Factor name**	**Statistic value**	**P-value**
Fig. 3d		**PL mPFC AAV-control**		
	Kolmogorov-Smirnov	Firing rate baseline	$K\text{-}S = 0.19$	n.s.
		Firing rate CNO	$K\text{-}S = 0.18$	n.s.
	Paired t-test	Firing rate	$t = 1.50$	n.s.
		**PL mPFC AAV-hM4Di**		
	Kolmogorov-Smirnov	Firing rate baseline	$K\text{-}S = 0.19$	n.s.
		Firing rate CNO	$K\text{-}S = 0.22$	n.s.
	Paired t-test	Firing rate	$t = 2.94$	$P < 0.05$
Fig. 3f		**NAc AAV-control**		
		mEPSCs frequency CNO	$K\text{-}S = 0.22$	n.s.
	Paired t-test	mEPSCs frequency	$t = -0.22$	n.s.
		**NAc AAV-hM4Di**		
		mEPSCs frequency CNO	$K\text{-}S = 0.28$	n.s.
	Paired t-test	mEPSCs frequency	$t = 3.48$	$P < 0.05$
Fig. 3h	Repeated measures ANOVA	**FR1 (Sessions 1-2)**		
		Treatment	$F_{(1,32)} = 0.15$	n.s
		Sessions	$F_{(8, 256)} = 16.11$	$P < 0.001$
		Treatment x Sessions	$F_{(8, 256)} = 0.01$	n.s
		**FR5 (Sessions 3-9)**		
		Treatment	$F_{(1,32)} = 0.22$	n.s
		Sessions	$F_{(8, 256)} = 21.62$	$P < 0.001$
		Treatment x Sessions	$F_{(8, 256)} = 1.64$	n.s
		**FR5 (Sessions 10-20)**		
		Treatment	$F_{(1,15)} = 0.55$	n.s
		Sessions	$F_{(10,150)} = 6.37$	$P < 0.001$
		Treatment x Sessions	$F_{(10,150)} = 0.91$	n.s
Fig. 3i-k	Kolmogorov-Smirnov	Persistence to response	$K\text{-}S = 0.09$	n.s
		Motivation	$K\text{-}S = 0.11$	n.s
		Compulsivity	$K\text{-}S = 0.17$	$P < 0.05$
	t-test (Equal variances assumed)	Persistence to response	$t = 1.16$	n.s.
		Motivation	$t = -0.56$	n.s.
	U Mann-Whitney	Compulsivity	$U = 48.50$	$P < 0.01$
Fig. 3l	Chi square	Treatment	$C\text{-}S = 17.60$	$P < 0.001$
Fig. 3m-o	Pearson correlation	**Saline**		
		Non-reinforced active responses in 10 min and addiction criteria	$r = 0.50$	n.s.
		Breaking point in 5h and addiction criteria	$r = 0.46$	n.s.
		Compulsivity and addiction criteria	$r = 0.60$	$P < 0.05$
		**CNO**		
		Non-reinforced active responses in 10 min and addiction criteria	$r = 0.57$	$P < 0.01$
		Breaking point in 5h and addiction criteria	$r = 0.51$	$P < 0.05$
		Compulsivity and addiction criteria	$r = 0.45$	$P < 0.05$
Supplementary Fig. 6b		**PL mPFC AAV-control**		
	Kolmogorov-Smirnov	Resistance baseline	$K\text{-}S = 0.19$	n.s.
		Resistance CNO	$K\text{-}S = 0.17$	n.s.
	Paired t-test	Resistance	$t = -1.50$	n.s.

Supplementary Fig. 6b	Kolmogorov-Smirnov	**PL mPFC AAV-hM4Di**		
		Resistance baseline	*K-S* = 0.19	n.s.
		Resistance CNO	*K-S* = 0.19	n.s.
	Paired t-test	Resistance	*t* = 3.58	$P < 0.01$
Supplementary Fig. 6d	Kolmogorov-Smirnov	**PL mPFC AAV-control**		
		Rheobase baseline	*K-S* = 0.17	n.s.
		Rheobase CNO	*K-S* = 0.20	n.s.
	Paired t-test	Rheobase	*t* = -0.37	n.s.
	Kolmogorov-Smirnov	**PL mPFC AAV-hM4Di**		
		Rheobase baseline	*K-S* = 0.17	n.s.
		Rehobase CNO	*K-S* = 0.15	n.s.
	Paired t-test	Rehobase	*t* = -4.05	$P < 0.01$
Supplementary Fig. 6f		**NAc AAV-control**		
	Kolmogorov-Smirnov	mEPSCs amplitude CNO	*K-S* = 0.32	$P < 0.05$
	Wilcoxon test	mEPSCs amplitude	*Z* = -0.42	n.s.
		**NAc AAV-hM4Di**		
	Kolmogorov-Smirnov	mEPSCs Vm CNO	*K-S* = 0.19	n.s.
	Paired t-test	mEPSCs Vm	*t* = 0.27	n.s.
Supplementary Fig. 6g		**NAc AAV-control**		
	Kolmogorov-Smirnov	mEPSCs Vm CNO	*K-S* = 0.20	n.s.
	Paired t-test	mEPSCs Vm	*t* = -1.48	n.s.
		**NAc AAV-hM4Di**		
	Kolmogorov-Smirnov	mEPSCs Vm CNO	*K-S* = 0.31	$P < 0.05$
	Wilcoxon test	mEPSCs Vm	*Z* = -1.48	n.s.
Supplementary Fig. 6h-j	Repeated measures ANOVA	**Body Weight**		
		Treatment	$F_{(1,32)} = 0.65$	n.s
		Weeks	$F_{(3,96)} = 43.38$	$P < 0.001$
		Treatment x Weeks	$F_{(3,96)} = 0.41$	n.s
		**Food Intake**		
		Treatment	$F_{(1,16)} = 0.44$	n.s
		Weeks	$F_{(3,48)} = 4.68$	$P < 0.001$
		Treatment x Weeks	$F_{(3,48)} = 0.71$	n.s
		**Kinetics of total activity**		
		Treatment	$F_{(1,17)} = 0.07$	n.s
		Time	$F_{(11,187)} = 9.04$	$P < 0.001$
		Treatment x Time	$F_{(11,187)} = 1.14$	n.s
Supplementary Fig. 6k-m	Kolmogorov-Smirnov	Persistence to response	*K-S* = 0.09	n.s
		Motivation	*K-S* = 0.12	n.s
		Compulsivity	*K-S* = 0.17	$P < 0.05$
	t-test (Equal variances assumed)	**Persistence to response**		
		Saline NA vs Saline A	*t* = -1.69	n.s.
		Saline NA vs CNO NA	*t* = 0.05	n.s.
		Saline NA vs CNO A	*t* = -2.97	$P < 0.01$
		Saline A vs CNO NA	*t* = 1.91	n.s.
		Saline A vs CNO A	*t* = -0.19	n.s.
		CNO NA vs CNO A	*t* = -3.23	$P < 0.01$
		**Motivation**		
		Saline NA vs Saline A	*t* = -3.50	$P < 0.01$
		Saline NA vs CNO NA	*t* = 0.92	n.s.
		Saline NA vs CNO A	*t* = -1.49	n.s.
		Saline A vs CNO NA	*t* = 4.32	$P < 0.01$
		Saline A vs CNO A	*t* = 2.30	$P < 0.05$
		CNO NA vs CNO A	*t* = -2.46	$P < 0.05$

Supplementary Fig. 6k-m	U Mann-Whitney	**Compulsivity**		
		Saline NA vs Saline A	*U* = 5.50	n.s.
		Saline NA vs CNO NA	*U* = 27.00	*P* < 0.05
		Saline NA vs CNO A	*U* = 10.0	*P* < 0.01
		Saline A vs CNO NA	*U* = 8.0	n.s.
		Saline A vs CNO A	*U* = 3.50	n.s.
		CNO NA vs CNO A	*U* = 43.00	n.s.

Supplementary Table 5. Statistical details of experiments shown in Fig. 4 and Supplementary Figure 7

Drd2 gene expression is upregulated in mPFC of addicted mice				
**Figure number**	**Statistical analysis**	**Factor name**	**Statistic value**	**P-value**
Fig. 4a-d	Kolmogorov-Smirnov	Persistence to response	$K\text{-}S = 0.26$	$P < 0.01$
		Motivation	$K\text{-}S = 0.17$	n.s.
		Compulsivity	$K\text{-}S = 0.29$	$P < 0.001$
		Pellets intake in the last FR5 session	$K\text{-}S = 0.14$	n.s .
	U Mann-Whitney	**Persistence to response**		
		WT NA vs WT A	$U = 0.00$	$P < 0.01$
		WT NA vs Glu-CB1-KO NA	$U = 7.50$	n.s.
		WT NA vs Glu-CB1-KO A	$U = 3.50$	n.s.
		WT A vs Glu-CB1-KO NA	$U = 0.00$	$P < 0.01$
		WT A vs Glu-CB1-KO A	$U = 9.00$	n.s.
		Glu-CB1-KO NA vs Glu-CB1-KO A	$U = 2.50$	$P < 0.05$
	t-test (Equal variances assumed)	**Motivation**		
		WT NA vs WT A	$t = -3.52$	$P < 0.01$
		WT NA vs Glu-CB1-KO NA	$t = 1.06$	n.s.
		WT NA vs Glu-CB1-KO A	$t = - 3.70$	$P < 0.01$
		WT A vs Glu-CB1-KO NA	$t = 4.20$	$P < 0.01$
		WT A vs Glu-CB1-KO A	$t = -0.52$	n.s.
		Glu-CB1-KO NA vs Glu-CB1-KO A	$t = 4.33$	$P < 0.01$
	U Mann-Whitney	**Compulsivity**		
		WT NA vs WT A	$U = 2.50$	$P < 0.05$
		WT NA vs Glu-CB1-KO NA	$U = 12.50$	n.s.
		WT NA vs Glu-CB1-KO A	$U = 0.00$	$P < 0.01$
		WT A vs Glu-CB1-KO NA	$U = 2.50$	$P < 0.05$
		WT A vs Glu-CB1-KO A	$U = 10.50$	n.s.
		Glu-CB1-KO NA vs Glu-CB1-KO A	$U = 0.00$	$P < 0.01$
	t-test (Equal variances assumed)	**Pellets intake in the last FR5 session**		
		WT NA vs WT A	$t = -1.20$	n.s.
		WT NA vs Glu-CB1-KO NA	$t = 2.81$	$P < 0.05$
		WT NA vs Glu-CB1-KO A	$t = 1.10$	n.s.
		WT A vs Glu-CB1-KO NA	$t = 4.47$	$P < 0.01$
		WT A vs Glu-CB1-KO A	$t = 2.51$	$P < 0.05$
		Glu-CB1-KO NA vs Glu-CB1-KO A	$t = 2.20$	n.s.
Fig. 4f	Kolmogorov-Smirnov	*Drd2*	$K\text{-}S = 0.27$	$P < 0.01$
		Adora2a	$K\text{-}S = 0.27$	$P < 0.001$
		Gpr88	$K\text{-}S = 0.22$	$P < 0.05$
		Drd1	$K\text{-}S = 0.25$	$P < 0.01$
	t-test (Equal variances assumed)	*Drd2*	$t = -2.56$	$P < 0.05$
		Adora2a	$t = -2.30$	$P < 0.05$
	t-test (Equal variances not assumed)	*Gpr88*	$t = -2.11$	$P < 0.05$
		Drd1	$t = -2.51$	$P < 0.05$
Fig. 4h	Kolmogorov-Smirnov	*Cnr1*	$K\text{-}S = 0.15$	n.s.
		Fos	$K\text{-}S = 0.33$	$P < 0.001$
		Npas4	$K\text{-}S = 0.18$	n.s.
	t-test (Equal variances assumed)	*Cnr1*	$t = 7.78$	$P < 0.001$
	U Mann-Whitney	*Fos*	$U = 22$	$P < 0.05$

Fig. 4h	t-test (Equal variances assumed)	*Npas4*	*t* = 1.49	n.s.
Supplementary Fig. 7c-e	Kolmogorov-Smirnov	*Tbp*	*K-S* = 0.10	n.s.
		Usp11	*K-S* = 0.17	n.s.
		Actb	*K-S* = 0.17	n.s.
	t-test (Equal variances assumed)	*Tbp*	*t* = -0.27	n.s.
		Usp11	*t* = 0.94	n.s.
		Actb	*t* = 0.38	n.s.

Supplementary Table 6. Statistical details of experiments shown in Fig. 5 and Supplementary Figure 8-10

***Drd2* overexpression in PL-NAc core pathway promotes compulsivity**				
**Figure number**	**Statistical analysis**	**Factor name**	**Statistic value**	***P*- value**
Fig. 5d		**PL mPFC AAV-control**		
	Kolmogorov-Smirnov	Firing rate baseline	*K-S* = 0.14	n.s.
		Firing rate Quinpirole	*K-S* = 0.24	n.s.
	Paired t-test	Firing rate	t = -0.37	n.s.
		**PL mPFC AAV-D2R**		
	Kolmogorov-Smirnov	Firing rate baseline	*K-S* = 0.30	$P < 0.05$
		Firing rate Quinpirole	*K-S* = 0.30	$P < 0.05$
	Paired t-test	Firing rate	Z = -2.41	$P < 0.05$
Fig. 5f		**NAc AAV-control**		
		mEPSCs frequency Quinpirole	*K-S* = 0.21	n.s.
	Paired t-test	mEPSCs frequency	t = 0.18	n.s.
		**NAc AAV-D2R**		
		mEPSCs frequency Quinpirole	*K-S* = 0.27	$P < 0.05$
	Wilcoxon test	mEPSCs frequency	Z = -2.80	$P < 0.01$
Fig. 5h	Repeated measures ANOVA	**FR1 (Sessions 1-2)**		
		AAV PL	$F_{(1,23)} = 0.01$	n.s
		Sessions	$F_{(1,23)} = 12.19$	$P < 0.001$
		AAV PL x Sessions	$F_{(1,23)} = 0.72$	n.s
		**FR5 (Sessions 3-9)**		
		AAV PL	$F_{(1,23)} = 0.07$	n.s
		Sessions	$F_{(6,184)} = 25.58$	$P < 0.001$
		AAV PL x Sessions	$F_{(6,184)} = 0.43$	n.s
		**FR5 (Sessions 10-24)**		
		AAV PL	$F_{(1,23)} = 0.25$	n.s
		Sessions	$F_{(14,322)} = 20.23$	$P < 0.001$
		AAV PL x Sessions	$F_{(14,322)} = 0.45$	n.s
Fig. 5i-k	Kolmogorov-Smirnov	Persistence to response	*K-S* = 0.19	$P < 0.05$
		Motivation	*K-S* = 0.16	n.s
		Compulsivity	*K-S* = 0.17	n.s
	U Mann-Whitney	Persistence to response	U = 47.00	n.s.
	t-test (Equal variances assumed)	Motivation	t = -0.52	n.s.
		Compulsivity	t = -2.77	$P < 0.05$
Fig. 5l	Chi square	AAV PL	*C-S* = 8.57	$P < 0.001$
Fig. 5m-o	Pearson correlation	**Control**		
		Non-reinforced active responses in 10 min and addiction criteria	r = 0.34	$P < 0.05$
		Breaking point in 5h and addiction criteria	r = 0.66	$P < 0.05$
		Compulsivity and addiction criteria	r = 0.06	n.s.
		**D2R**		
		Non-reinforced active responses in 10 min and addiction criteria	r = -0.03	$P < 0.05$
		Breaking point in 5h and addiction criteria	r = 0.40	n.s.
		Compulsivity and addiction criteria	r = 0.40	n.s.
Supplementary Fig. 8d		**qPCR**		
	Kolmogorov-Smirnov	Drd2 mRNA	*K-S* = 0.23	$P < 0.05$
	U Mann-Whitney	Drd2 mRNA	U = 0.00	$P < 0.01$

Supplementary Fig. 9b		**PL mPFC AAV-control**		
	Kolmogorov-Smirnov	Resistance baseline	*K-S* = 0.27	n.s.
		Resistance Quinpirole	*K-S* = 0.16	n.s.
	Paired t-test	Resistance	*t* = 0.23	n.s.
		**PL mPFC AAV-D2R**		
	Kolmogorov-Smirnov	Resistance baseline	*K-S* = 0.18	n.s.
		Resistance Quinpirole	*K-S* = 0.24	n.s.
	Paired t-test	Resistance	*t* = 2.79	*P* < 0.05
Supplementary Fig. 9d		**PL mPFC AAV-control**		
	Kolmogorov-Smirnov	Rheobase baseline	*K-S* = 0.25	n.s.
		Rheobase Quinpirole	*K-S* = 0.38	*P* < 0.01
	Wilcoxon test	Rheobase	*Z* = -0.70	n.s.
		**PL mPFC AAV-D2R**		
	Kolmogorov-Smirnov	Rheobase baseline	*K-S* = 0.35	*P* < 0.01
		Rheobase Quinpirole	*K-S* = 0.34	*P* < 0.01
	Wilcoxon test	Rheobase	*Z* = -2.67	*P* < 0.01
Supplementary Fig. 9e		**NAc AAV-control**		
	Kolmogorov-Smirnov	mEPSCs amplitude CNO	*K-S* = 0.26	n.s.
	Paired t-test	mEPSCs amplitude	*t* = -0.15	n.s.
		**NAc AAV-D2R**		
	Kolmogorov-Smirnov	mEPSCs Vm CNO	*K-S* = 0.20	n.s.
	Paired t-test	mEPSCs Vm	*t* = 1.75	n.s.
Supplementary Fig. 9f		**NAc AAV-control**		
	Kolmogorov-Smirnov	mEPSCs Vm CNO	*K-S* = 0.29	n.s.
	Paired t-test	mEPSCs Vm	*t* = -1.15	n.s.
		**NAc AAV-D2R**		
	Kolmogorov-Smirnov	mEPSCs Vm CNO	*K-S* = 0.12	*P* < 0.05
	Paired t-test	mEPSCs Vm	*t* = 1.48	n.s.
Supplementary Fig. 9h		**PL mPFC AAV-control**		
	Kolmogorov-Smirnov	Firing rate baseline	*K-S* = 0.17	n.s.
		Firing rate Dopamine	*K-S* = 0.12	n.s.
	Paired t-test	Firing rate	*t* = 1.13	n.s.
		**PL mPFC AAV-D2R**		
	Kolmogorov-Smirnov	Firing rate baseline	*K-S* = 0.35	*P* < 0.01
		Firing rate Dopamine	*K-S* = 0.35	*P* < 0.01
	Wilcoxon test	Firing rate	*Z* = -2.01	*P* < 0.05
Supplementary Fig. 9j		**PL mPFC AAV-control**		
	Kolmogorov-Smirnov	Resistance baseline	*K-S* = 0.19	n.s.
		Resistance Dopamine	*K-S* = 0.20	n.s.
	Paired t-test	Resistance	*t* = -1.37	n.s.
		**PL mPFC AAV-D2R**		
	Kolmogorov-Smirnov	Resistance baseline	*K-S* = 0.16	n.s.
		Resistance Dopamine	*K-S* = 0.21	n.s.
	Paired t-test	Resistance	*t* = 6.00	*P* < 0.001
Supplementary Fig. 9l		**PL mPFC AAV-control**		
	Kolmogorov-Smirnov	Rheobase baseline	*K-S* = 0.18	n.s.
		Rheobase Dopamine	*K-S* = 0.16	n.s.
	Paired t-test	Rheobase	*Z* = -0.70	n.s.
		**PL mPFC AAV-D2R**		
	Kolmogorov-Smirnov	Rheobase baseline	*K-S* = 0.33	*P* < 0.01
		Rheobase Dopamine	*K-S* = 0.15	n.s.
	Wilcoxon test	Rheobase	*Z* = -2.80	*P* < 0.01

Supplementary Fig. 10a-c	Kolmogorov-Smirnov	Persistence to response	*K-S* = 0.19	P < 0.05
		Motivation	*K-S* = 0.16	n.s
		Compulsivity	*K-S* = 0.17	n.s
	U Mann-Whitney	**Persistence to response**		
		Control NA vs Control A	*U* = 1.00	n.s.
		Control NA vs D2R NA	*U* = 33.00	n.s.
		Control NA vs D2R A	*U* = 13.00	n.s.
		Control A vs D2R NA	*U* = 1.00	n.s.
		Control A vs D2R A	*U* = 0.00	n.s.
		D2R NA vs D2R A	*U* = 14.50	n.s.
	T-test (Equal variances assumed)	**Motivation**		
		Control NA vs Control A	*t* = -2.66	*P* < 0.05
		Control NA vs D2R NA	*t* = -0.20	n.s.
		Control NA vs D2R A	*t* = -2.80	*P* < 0.05
		Control A vs D2R NA	*t* = 1.34	n.s.
		Control A vs D2R A	*t* = 2.63	n.s.
		D2R NA vs D2R A	*t* = -1.37	n.s.
		**Compulsivity**		
		Control NA vs Control A	*t* = 0.57	n.s.
	t-test (Equal variances	Control NA vs D2R NA	*t* = -2.27	*P* < 0.05
	t-test (Equal variances assumed)	Control NA vs D2R A	*t* = -2.70	*P* < 0.05
		Control A vs D2R NA	*t* = -1.05	n.s.
		Control A vs D2R A	*t* = -0.94	n.s.
		D2R NA vs D2R A	*t* = -0.96	n.s.
Supplementary Fig. 10d-f	Repeated measures ANOVA	**Body Weight**		
		AAV PL	$F_{(1,23)} = 0.00$	n.s
		Weeks	$F_{(3,69)} = 11.29$	*P* < 0.001
		AAV PL x Weeks	$F_{(3,69)} = 0.53$	n.s
		**Food Intake**		
		AAV PL	$F_{(1,23)} = 1.24$	n.s
		Weeks	$F_{(3,69)} = 4.23$	*P* < 0.05
		AAV PL x Weeks	$F_{(3,69)} = 0.65$	n.s
		**Kinetics of total activity**		
		AAV PL	$F_{(1,22)} = 1.33$	n.s
		Time	$F_{(11,242)} = 28.15$	*P* < 0.001
		AAV PL x Time	$F_{(11,242)} = 1.09$	n.s

Supplementary Table 7. List of differentially expressed upregulated genes between addicted and non-addicted mice

	Gene ID	Mean read counts (Non-addicted)	Mean read counts (Addicted mice)	Log2 Fold Change	p-value	Adjusted p-value
1	*Drd2*	56.6018054	160.8662034	1.506941284	3.58E-38	7.74E-34
2	*Ecel1*	77.64410956	197.9691909	1.350327543	2.43E-35	2.62E-31
3	*Adora2a*	168.7859333	369.9244864	1.132036126	7.78E-35	5.60E-31
4	*Syndig1l*	113.1561661	243.6965918	1.106770872	1.60E-28	5.77E-25
5	*Gpr88*	687.0699975	1234.927885	0.845897805	7.12E-27	2.20E-23
6	*Lrrc10b*	142.2415661	282.6513973	0.990680716	3.03E-25	6.54E-22
7	*Gpr6*	45.39651027	111.9463932	1.302154743	1.03E-24	2.02E-21
8	*Drd1*	459.6024675	798.510399	0.796924652	1.22E-22	2.02E-19
9	*Ppp1r1b*	1067.424527	1790.865452	0.746522884	6.36E-22	9.81E-19
10	*Rgs9*	885.8954502	1478.582089	0.739005989	1.26E-21	1.81E-18
11	*Sh3rf2*	34.41122537	84.85534273	1.302126232	6.75E-21	8.09E-18
12	*Slc5a7*	39.33052903	91.35561709	1.215843845	2.13E-19	2.42E-16
13	*Penk*	783.6319634	1248.165147	0.671560683	1.88E-18	1.76E-15
14	*Pde10a*	2323.670729	3646.993164	0.650301846	3.85E-18	3.46E-15
15	*Hspa1b*	203.4111568	349.4882397	0.780845099	1.25E-17	1.08E-14
16	*Prkcd*	205.4579881	341.5377357	0.733201564	2.10E-15	1.68E-12
17	*Chat*	17.06759663	45.10123577	1.401907044	1.00E-14	7.22E-12
18	*Hspa1a*	163.1024926	271.3450752	0.734349895	1.63E-14	1.14E-11
19	*Glp1r*	18.56124092	47.49588207	1.355509271	4.50E-14	2.78E-11
20	*Cd4*	24.91278103	55.88781781	1.165647765	5.74E-13	3.02E-10
21	*Dlk1*	23.52272328	52.46635936	1.157337591	4.78E-12	2.29E-09
22	*Thbs4*	94.63048848	155.0216073	0.712092339	9.42E-11	3.51E-08
23	*Clic6*	37.33055701	67.52640259	0.855094666	1.50E-09	4.37E-07
24	*Foxj1*	73.1750867	118.3823861	0.694029985	4.57E-09	1.17E-06
25	*Serpina9*	48.4000399	81.37900834	0.749648463	1.01E-08	2.25E-06
26	*Top2a*	38.44465301	67.87621159	0.820123087	1.12E-08	2.44E-06
27	*Mid1*	110.7074736	169.3693633	0.613420316	8.95E-08	1.57E-05
28	*4932418E24Rik*	56.84999912	87.98192951	0.630046914	6.01E-07	8.31E-05
29	*Spint1*	35.135819	55.35830752	0.655857308	1.33E-05	1.37E-03
30	*Ttc21a*	24.7930007	40.92264726	0.722966584	4.20E-05	3.61E-03
31	*Gpr101*	33.72793119	51.42627896	0.60856194	7.37E-05	5.81E-03

The differentially expressed genes have a fold change > 1.5 fold, P< 0.01 and average read counts > 40.

Supplementary Table 8. List of differentially expressed downregulated genes between addicted and non-addicted mice

	GeneID	Mean read counts (Non-addicted)	Mean read counts (Addicted mice)	Log2 Fold Change	p-value	Adjusted p-value
1	*Dct*	109.3353486	39.12531022	-1.48258581	3.62E-29	1.56E-25
2	*Tyrp1*	45.84567001	20.33049298	-1.173140283	1.64E-11	6.96E-09
3	*H2-Eb1*	55.9586155	26.10119876	-1.100244201	7.17E-12	3.29E-09
4	*Maff*	55.44815072	25.93006392	-1.096513583	9.39E-11	3.51E-08
5	*Flnc*	194.7909403	91.29721647	-1.093283799	3.02E-23	5.43E-20
6	*H2-Aa*	57.47972395	27.76640275	-1.049712851	1.49E-11	6.57E-09
7	*Slc47a1*	72.18625099	34.90031642	-1.048483963	3.39E-13	1.87E-10
8	*Slc22a6*	242.0156839	117.7981031	-1.038784237	4.62E-26	1.11E-22
9	*Slc13a4*	339.8016262	172.4680299	-0.9783638	1.24E-26	3.35E-23
10	*Cd74*	122.1143353	64.54366117	-0.919885251	5.78E-15	4.30E-12
11	*Cdh1*	48.69093854	25.85653509	-0.91312435	9.11E-09	2.05E-06
12	*H2-Ab1*	49.11280784	26.13346893	-0.910200666	2.85E-08	5.65E-06
13	*Crabp2*	50.73144363	27.01937017	-0.908886169	2.21E-08	4.54E-06
14	*Wnt6*	65.68513716	35.32180676	-0.895007822	3.93E-10	1.26E-07
15	*Ptgds*	10011.69697	5410.76253	-0.8877827	2.72E-34	1.47E-30
16	*Aldh1a2*	232.6670426	126.0162471	-0.884657116	4.61E-19	4.98E-16
17	*Cyp1b1*	128.3799703	70.54725679	-0.863758243	1.57E-13	9.17E-11
18	*Fmod*	342.9951115	189.2917796	-0.857576255	2.06E-21	2.61E-18
19	*Slc6a12*	46.43178292	25.9133909	-0.841414868	3.46E-07	5.12E-05
20	*Mpzl2*	48.36084501	27.11436907	-0.834781852	1.68E-07	2.72E-05
21	*Foxd1*	47.4052797	26.73721804	-0.826198383	3.19E-07	4.79E-05
22	*Fgl2*	94.23828715	53.16059862	-0.825955967	1.01E-10	3.69E-08
23	*Aebp1*	511.1484958	290.7724936	-0.813851675	1.73E-21	2.33E-18
24	*Trdn*	43.31476733	24.6837131	-0.811299536	2.57E-06	3.11E-04
25	*S100a5*	54.44701074	31.65568349	-0.782388298	2.51E-07	3.87E-05
26	*Gjb2*	210.8534879	124.3091354	-0.762308563	3.04E-14	2.05E-11
27	*Slc6a20a*	341.0821537	201.5841543	-0.758737032	8.88E-17	7.37E-14
28	*Ppm1j*	71.79725655	42.46683504	-0.757592126	8.15E-08	1.46E-05
29	*Prg4*	49.23929615	29.14340148	-0.756640867	4.31E-06	5.00E-04
30	*Crispld2*	73.28988348	43.39205162	-0.756183271	5.27E-08	9.72E-06
31	*Sphk1*	94.2171474	55.80844351	-0.755506242	1.82E-09	5.09E-07
32	*Ptgis*	70.5704174	41.96466995	-0.749888309	6.50E-08	1.18E-05
33	*Igfbp2*	723.1413825	430.3393791	-0.748802875	5.45E-19	5.60E-16
34	*Ogn*	216.4040394	128.8282767	-0.748278142	4.99E-15	3.85E-12
35	*Thbs1*	127.2322628	76.07600904	-0.741951078	3.35E-10	1.11E-07
36	*8430408G22Rik*	41.1893236	24.74797268	-0.734960088	6.25E-05	5.06E-03
37	*Igf2*	757.0006486	461.1674896	-0.715003724	1.64E-18	1.61E-15
38	*Svep1*	128.6590445	79.28488577	-0.698435106	7.11E-10	2.19E-07
39	*Shisa8*	335.4081077	206.8997684	-0.696985533	1.98E-13	1.12E-10
40	*Gpnmb*	125.3062407	77.4453185	-0.69420833	1.61E-09	4.64E-07
41	*Col13a1*	49.8281176	30.98051054	-0.685599154	1.47E-05	1.49E-03

42	*Adamtsl3*	122.068845	76.0663507	-0.682364737	4.36E-09	1.15E-06
43	*Npffr2*	53.16796688	33.22963043	-0.678087052	2.07E-05	2.01E-03
44	*Trh*	180.8000167	113.215686	-0.675320954	1.56E-10	5.35E-08
45	*Lrrc32*	85.01959703	54.10889665	-0.651929598	1.47E-06	1.90E-04
46	*Mrc2*	314.9115026	200.8114313	-0.649105057	1.11E-12	5.63E-10
47	*Slc6a13*	380.1193637	242.8111063	-0.646618107	1.28E-13	7.68E-11
48	*Osr1*	46.57377963	29.81019679	-0.643712068	3.29E-05	2.97E-03
49	*Itih2*	157.7098947	101.3681152	-0.637669246	1.05E-09	3.11E-07
50	*F13a1*	94.48726878	60.76323428	-0.636921291	4.31E-07	6.20E-05
51	*Myh11*	576.2763205	372.2260338	-0.630581778	4.01E-14	2.62E-11
52	*Adamts19*	297.2769742	192.7192529	-0.625307016	1.76E-11	7.29E-09
53	*Aqp1*	119.8855643	77.87373835	-0.622449161	2.96E-08	5.80E-06
54	*Col3a1*	129.4019559	84.4385093	-0.615886409	2.67E-08	5.35E-06
55	*Postn*	68.0255026	44.54221997	-0.610902247	1.39E-05	1.42E-03
56	*Fosb*	340.5819776	223.0156766	-0.610856969	6.30E-10	1.97E-07
57	*Myoc*	316.2675853	207.1324891	-0.610591839	4.82E-11	1.89E-08
58	*Fibin*	71.39925111	46.88618106	-0.606746168	1.33E-05	1.37E-03
59	*Acta2*	452.4502944	297.5415379	-0.60466822	1.91E-12	9.35E-10
60	*Mgp*	275.7014721	181.6124811	-0.602243617	1.06E-10	3.82E-08
61	*Frmd7*	624.8698447	411.6566515	-0.602114182	4.40E-13	2.37E-10
62	*Hpse*	44.43219398	29.27935274	-0.601721719	2.79E-04	1.77E-02
63	*Shisa3*	178.8309727	117.915563	-0.600842483	6.72E-09	1.56E-06
64	*Ctxn3*	48.30369443	31.91896819	-0.59771952	2.84E-04	1.78E-02
65	*Mrc1*	189.9957818	125.5506139	-0.597698306	4.63E-09	1.18E-06
66	*Vipr2*	183.3122265	121.2102772	-0.596790986	1.56E-08	3.34E-06
67	*Nupr1*	80.02454085	53.11965367	-0.591196754	5.76E-06	6.44E-04
68	*Cyp26b1*	130.0908159	86.56031261	-0.587741501	1.02E-07	1.78E-05
69	*Tspan11*	41.8793429	27.89117847	-0.586429913	4.75E-04	2.71E-02
70	*Emilin1*	93.11204912	62.14299767	-0.583376035	3.76E-06	4.39E-04

The differentially expressed genes have a fold change > 1.5 fold, P<0.01 and average read counts > 40.

Supplementary Table 9. List of differentially expressed upregulated genes between WT and Glu-CB1-KO mice

	GeneID	Mean read counts (WT)	Mean read counts (Glu-CB1-KO)	Log2 Fold Change	p-value	Adjusted p-value
1	*Spaca1*	2.5818864	413.8064362	7.324386749	0	0
2	*Dnah6*	92.49892452	244.4641197	1.402114238	1.74E-40	1.88E-36
3	*Gm3279*	6.618737271	44.03495206	2.734021185	1.53E-34	1.10E-30
4	*Nmbr*	97.70806547	178.6976333	0.870970965	3.44E-15	7.43E-12
5	*Ndst4*	448.7878526	703.0002903	0.647491658	6.58E-14	1.18E-10
6	*Strip2*	979.6659127	1482.008864	0.597192329	7.73E-14	1.28E-10
7	*Dnah11*	406.0169287	628.0292637	0.629291903	9.22E-14	1.42E-10
8	*Shisa8*	215.4570524	339.2279093	0.654854547	6.90E-12	8.28E-09
9	*Gcnt1*	178.8801351	276.1143757	0.62627282	9.30E-11	8.03E-08
10	*Scgn*	95.00994668	156.610498	0.72103046	1.69E-10	1.40E-07
11	*Snora68*	191.4693955	293.7446511	0.617448771	3.17E-10	2.45E-07
12	*Snora78*	174.099957	264.3890279	0.60274646	1.16E-09	7.62E-07
13	*Htra4*	45.3640974	78.59586393	0.792902439	1.02E-08	5.23E-06
14	*Snora70*	80.99417938	124.2918029	0.617841015	2.11E-07	7.46E-05
15	*Snord8*	72.76571116	111.7793	0.61932236	5.29E-07	1.73E-04
16	*S100a5*	33.96215186	54.16036354	0.673309551	7.76E-06	1.78E-03
17	*Snora31*	40.12685021	61.55568439	0.617324174	4.39E-05	7.45E-03
18	*Rhcg*	28.99370161	44.15639058	0.606882715	5.05E-04	5.02E-02

The differentially expressed genes have a fold change > 1.5 fold, P<0.01 and average read counts > 40.

Supplementary Table 10. List of differentially expressed downregulated genes between WT and Glu-CB1-KO mice

	GeneID	Mean read counts (WT)	Mean read counts (Glu-CB1-KO)	Log2 Fold Change	p-value	Adjusted p-value
1	*Egr2*	219.1145629	111.0433213	-0.980562748	6.97E-20	3.20E-16
2	*Neurod6*	1184.883595	702.1656958	-0.754861912	7.41E-20	3.20E-16
3	*Cnr1*	4713.295932	2917.140044	-0.692181618	7.31E-19	2.54E-15
4	*Fos*	695.9900747	406.8582808	-0.774540378	8.23E-19	2.54E-15
5	*Igf2*	742.0334482	446.5903835	-0.732532034	4.17E-17	1.13E-13
6	*Nr4a1*	1921.816765	1219.182576	-0.6565566	2.36E-16	5.67E-13
7	*Flnc*	180.1639633	97.67533014	-0.883244338	1.79E-14	3.51E-11
8	*Myh11*	564.7140441	363.6854509	-0.634829298	1.47E-12	1.98E-09
9	*Acta2*	446.5915513	287.4901399	-0.635443482	5.49E-12	6.97E-09
10	*Dusp1*	557.9952555	369.6287871	-0.594175736	9.38E-12	9.76E-09
11	*Fosb*	338.2003962	212.8635759	-0.667949245	9.44E-12	9.76E-09
12	*Adamts1*	333.3179029	210.9272924	-0.660153027	1.51E-11	1.48E-08
13	*Tagln*	340.9161734	226.7480564	-0.58832686	8.94E-10	6.43E-07
14	*Bgn*	229.3193043	146.8568425	-0.64294732	9.31E-10	6.48E-07
15	*Fmod*	314.3889502	207.1767176	-0.601688629	1.74E-09	1.07E-06
16	*Gjb2*	198.8190975	129.4017956	-0.619598704	1.25E-08	6.28E-06
17	*Maff*	52.53188811	26.21459745	-1.002823047	1.31E-08	6.42E-06
18	*Serping1*	142.336893	89.53989395	-0.668707135	1.37E-08	6.58E-06
19	*Dct*	90.51008653	54.33284849	-0.736253892	3.39E-08	1.46E-05
20	*Lrrc32*	84.98337226	50.71864912	-0.744664273	8.10E-08	3.30E-05
21	*Cd74*	111.1725017	71.52027186	-0.636375858	3.85E-07	1.32E-04
22	*Lrrc17*	46.24384681	26.44909033	-0.80604331	4.97E-06	1.29E-03
23	*Wnt6*	59.53434242	39.4657414	-0.593121276	1.32E-04	1.78E-02
24	*Cd6*	44.91230009	28.93651197	-0.63421958	2.98E-04	3.34E-02
25	*Osr1*	44.91944846	29.96953679	-0.583843428	8.69E-04	7.55E-02
26	*Cdh1*	44.06864161	28.96885318	-0.605249855	1.11E-03	8.78E-02

The differentially expressed genes have a fold change > 1.5 fold, P < 0.01 and average read counts > 40.

Supplementary Methods:

Animals. Male mice, aged 2-4 months, were housed individually in a temperature- and humidity-controlled laboratory conditions (21 ± 1ºC, 55 ± 10%) maintained with food and water *ad libitum.* Mice were tested during the dark phase of a reverse light cycle (lights off at 8.00 a.m and on at 20.00 p.m). Firstly, we used Glu-CB1-KO mice (CB1 floxed/floxed; Nex-Cre/+ mice), lacking CB_1R in dorsal telencephalic glutamatergic neurons, and their wild-type (WT) littermates in C57BL/6N background[1–4]. Secondly, we used Nex-Cre/+ mice expressing Cre recombinase in dorsal telencephalic glutamatergic neurons[5] and also WT JAX™ C57BL/6J (C57BL/6J) mice purchased from Charles River (France). All experimental protocols were performed in accordance with the guidelines of the European Communities Council Directive 2010/63/EU and approved by the local ethical committee (Comitè Ètic d'Experimentació Animal-Parc de Recerca Biomèdica de Barcelona, CEEA-PRBB, agreement N°9687). In agreement, maximal efforts were made to reduce the suffering and the number of mice used.

Behavioral experiments. *Operant behavior apparatus.* Mouse operant chambers (Model ENV-307A-CT, Med Associates, Georgia, VT, USA) were used for operant responding maintained by chocolate-flavored pellets. The operant chambers were equipped with two retractable levers, one randomly selected as the active lever and the other as the inactive. Pressing on the active lever resulted in a food pellet delivery paired with a stimulus-light (associated-cue), located above the active lever, and while pressing on the inactive lever had no consequences. A food dispenser equidistant between the two levers permitted the delivery of food pellets when required. The floor of the chambers was a grid floor that served to

deliver electric food shocks in the session of shock-test and served as a contextual cue in the session of shock-associated cue the day after the shock session. During the rest of self-administration sessions, a metal sheet with holes was placed above the grid floor. Thus, mice could discriminate between different contexts. The chambers were made of aluminum and acrylic and were housed in sound- and light-attenuated boxes equipped with fans to provide ventilation and white noise.

Food pellets. During the operant conditioning sessions, after active responding by lever pressing, animals received a 20 mg chocolate-flavored pellet, which is a highly palatable isocaloric pellet (TestDiet, Richmond, IN, USA). These pellets had a similar caloric value (3.44 kcal/g: 20.6% protein, 12.7% fat, 66.7% carbohydrate) of standard maintenance diet provided to mice in their home cage (3.52 kcal/g: 17.5% protein, 7.5% fat, 75% carbohydrate) with some slight differences in their composition: addition of chocolate flavor (2% pure unsweetened cocoa) and modification in the sucrose content. Indeed, although the carbohydrate content was similar in standard diet (75%) and in highly palatable isocaloric pellets (66.7%), the proportion of sucrose content in standard diet food was 8.3% and in highly palatable isocaloric pellets 50.1%.

Impulsivity. Non-reinforced active responses during the time-out periods (10 s) after each pellet delivery were measured as impulsivity-like behavior indicating the inability to stop a response once it is initiated[6]. The three consecutive days before the progressive ratio were considered.

Shock-induced conditioned suppression. Non-reinforced active responses during the following session after the shock-test were measured for the aversive associative learning.

Mice were placed in the self-administration chamber during 50 min with the same grid floor used during the shock-test. However, during this session, pressing the active lever had no consequences, no shock, no chocolate-flavored pellets and no cue-light.

Locomotor activity. Locomotor activity was evaluated by using individual locomotor activity boxes (10.8×20.3×18.6 cm, Imetronic, Pessac, France) equipped with infrared sensors to detect locomotor activity and an infrared plane to detect rearings. The boxes were provided with a removable cage, a sliding floor, a trough and a bottle. Mice were placed in the boxes during 2 h and the kinetics of the total activity (number of beam breaks) was recorded in blocks of 10 min.

Drugs. For the surgery procedure, ketamine hydrochloride (Imalgène; Merial Laboratorios S.A.) and medetomidine hydrochloride (Domtor; Esteve, Spain) were mixed and dissolved in sterile 0.9% physiological saline and administered intraperitoneally (i.p, 75 mg/kg and 1 mg/kg of body weight respectively) to anesthetize the mice. After surgery, anesthesia was reversed by a subcutaneous (s.c.) injection of atipamezole hydrochloride (Revertor; Virbac, Spain; 2.5 mg/kg of body weight) dissolved in sterile 0.9% physiological saline. In addition, mice received an i.p. injection of gentamicine (Genta-Gobens; LaboratoriosNormon, Spain; 1 mg/kg of body weight) and a s.c. injection of meloxicam (Metacam; BoehringerIngelheim, Rhein; 2 mg/kg of body weight) both dissolved in sterile 0.9% physiological saline.

For the activation of the inhibitory designer receptors exclusively activated by designer drugs (hM4Di-DREADD), clozapine N-oxide (CNO) (Enzo Life Sciences, NY) was administered using Alzet osmotic minipumps (Model 2004; alzet, Cupertino, CA) filled previously with CNO (diluted in 0.9% sterile saline; 5 mg/mL) or saline. The osmotic minipump was

implanted s.c. in the back of the mice under brief isofluorane anesthesia. Minipumps delivered a constant s.c. flow rate of 0.25 µl/h for 28 days.

For electrophysiological studies, we used WIN55,212-2 5 µM (Sigma-Aldrich, Spain), rimonabant 4 µM (Sanofi-Aventis, Spain), quinpirole hydrochloride 2 µM (Sigma-Aldrich, Spain) and dopamine hydrochloride 10 µM (Sigma-Aldrich, Spain).

RT-PCR validation. RNA was reverse transcribed using High Capacity RNA-to-cDNA kit (Applied Biosystems, 4390778). Primers for Taqman® Gene Expression Assay were purchased from Applied Biosystems. Real time PCR analysis was carried out with the following primers (gene name: probe code): *Actb*: Mm02619580_g1; *Adora2a*: Mm00802075_m1; *Cnr1*: Mm00432621_s1; *Drd2*: Mm00438545_m1; *Drd1*: Mm02620146_s1; *Fos*: Mm01302932_g1; *Gpr88*: Mm02620353_s1; *Npas4*: Mm01227866_g1; *Tbp*: Mm01277042_m1; *Usp11*: Mm00455198_m1. Relative expression of mRNAs was determined after normalization with housekeeping genes using the ΔΔCt method. We measured the gene expression of three different housekeeping genes (*Tbp*, *Usp11*, *actin)* in these samples using qPCR in order to verify that the expression of these genes was not affected by the operant model of food addiction used, as represented in Supplementary Material (Supplementary Figure 7c-e). Regarding the normalization of differentially expressed genes in qPCR validation, all validated differentially expressed genes were normalized using *Tbp* as housekeeping gene, although the same significant changes were also found using the other two housekeeping genes (*Usp11*, *actin*).

Furthermore, we measured the *Drd2* mRNA levels in the mPFC of control and D_2R-overexpressing mice by qPCR. qPCR analysis was carried out using designed specific

forward (TGCAGACCACCACCAACTAC) and reverse (GGAGGTGGTAGGTGAGTGGAAA) primers to target both mouse and human *Drd2* coding sequence and specific designed specific forward (CTCTGCTCCTCCCTGTTCC) and reverse (TCCCTAGACCCGTACAGTGC) primers for mouse *Gapdh* coding sequence. Designed primers and cDNA extracted from brain samples were used to carry the qPCR experiments following the same procedure and experimental conditions as described above, except that here SYBR Green methodoloy was applied (Life Technologies). Relative mRNA levels were determined after normalization with *Gapdh* as housekeeping gene using the ΔΔCt method.

Immunofluorescence studies. *Tissue preparation for immunofluorescence:* Mice were deeply anesthetized by i.p. injection (0.2 ml/10 g of body weight) of mixture of ketamine/medetomidine prior to intracardiac perfusion with 4% paraformaldehyde (PFA) in 0.1 M Na_2HPO_4/ 0.1 M NaH_2PO_4 buffer (PB), pH 7.5, delivered with a peristaltic pump at 30 ml per min for 2 min. Subsequently, brains were extracted and post-fixed with 4% PFA for 24 h and transferred to a solution of 30% sucrose at 4 °C. Coronal frozen sections (30 µm) of the PL and NAc core were obtained on a freezing microtome and stored in a 5% sucrose solution at 4°C until use.

Immunofluorescence: Free-floating slices were rinsed in 0.1 M PB, blocked in a solution containing 3% normal goat serum and 0.3% Triton X-100 in 0.1M PB (NGS-T-PB) at room temperature for 2 h, and incubated overnight at 4ºC in the same solution with the primary antibody to anti-Cre recombinase (1:500, mouse, MAB3120, Merck Millipore), anti-D_2R (1:1000, rabbit, D2R-Rb-Af96, Frontier Institute), anti-mVenus/GFP (1:1000, chicken,

ab13970, Abcam) or anti-GFP (1:500, rabbit, GTX20290, GeneTex).On the next day, after 3 rinses in 0.1 M PB, sections were incubated with the secondary antibody AlexaFluor-488 donkey anti-mouse (1:500, Life Technologies) or AlexaFluor-488 donkey anti-rabbit (1:500, Life Technologies) at room temperature in NGS-T-PB for 2 h. After incubation, sections were rinsed and mounted immediately after onto glass slides coated with gelatine in Fluoromount mounting medium.

Confocal microscope: The stained sections of the brain were analyzed with Leica TCS SP5 CFS (fixed stage) upright confocal microscope with two non-descanned HyD detectors.

Confocal imaging of mVenus and D_2R was performed using Visiscope 5-Elements spinning disk confocal system (Visitron Systems, Germany), equipped with Yokogawa CSU-W1 scan head and Prime BSI sCMOS camera (2048 x 2048 pixels, 6.5 µm pixel size, Photometrics). The laser lines of 488 nm and 561 nm were used for the fluorescence excitation and the fluorescence emission was filtered using filters 525/30 bandpass (Chroma) and 570 longpass (Chroma) for GFP and Alexa Fluor 546 respectively. The imaging was performed sequentially to minimize the spectral crosstalk. The large overview of the whole brain sections was imaged in tile scan mode using CFI Plan Apo Lambda 10x/ 0.45 NA air (Nikon) objective (effective pixel size of 665 nm). The zoomed-in regions were imaged using CFI Plan Apo VC 60x/ 1.2 NA Water immersion objective and an extra 2x magnification lens in the emission beam path (effective pixel size of 56 nm). The images were processed using the ImageJ analysis software

Fluorescence *in situ* hybridization. Double fluorescence *in situ* hybridization experiments were performed on coronal cortical sections and mPFC (PL and IL) and NAc were analyzed

using FITC labeled riboprobe for *Cre* recombinase and digoxigenin labeled riboprobe for dopamine D_2 receptor gene (*Drd2*) to detect *Cre*/*Drd2* double positive neurons to confirm the presence of both the endogenous *Drd2* mRNA and the mRNA of the injected *Cre* recombinase gene in the targeted cells. This cell population in the mPFC was analyzed in three AAV-retrograde-Cre injected WT C57BL/6J animals to determine overlapping expression *of Drd2* and of retrogradely travelled *Cre* recombinase-expressing virus. Slides with 6 parallel coronal sections of 3 animals, injected with AAV-retrograde–Cre were analyzed, containing NAc, PL and IL cortex (and striatum), the sections covering cortical region 2.10 – 1.18 mm anterior to bregma.

Adult WT C57BL/6J mice, injected in week 10-14 were sacrificed 4 weeks after injection by cervical dislocation. Brains were removed, snap-frozen on dry ice and stored at -80°C. After removing from -80°C, brains were mounted on Tissue Freezing Medium (Leica Biosystems) and 18 µm-thick coronal sections were cut from the frozen forebrain on a cryostat Leica CM3050 S, dried on a 42°C warming plate and stored at -20°C until used.

Both digoxigenin (DIG) and fluorescein isothiocyanate (FITC) labeled riboprobes were used. The DNA template for *Drd2* probe was originally generated by RT-PCR from cDNA derived from total mouse brain, previously reported[7]. GenBank accession number, primer sequences and length of probe are listed therein. For a riboprobe specific for *Cre* recombinase RNA, we isolated the stretch of cDNA from *Cre* recombinase sequence of the AAV-retrograde-Cre (Addgene vector AAV pmSyn1-EBFP-Cre) using a forward primer which contains at the 5' end also the EcoR1 recognition sequence as well as 5 nucleotides at the very 5'end (fw primer 5′-ACTATGAATTCCGAGTGATGAGGTTCGCAAG-3′) and the reverse primer containing at the 5' end the XhoI recognition sequence preceded by 5 nucleotides (rev primer

5’-AACTACTCGAGCCGGTATTGAAACTCCAGCG-3’) resulting in a 867 bp product. PCR products were cloned into pBluescript KS^- and used as templates for riboprobe synthesis as described. The identity of subcloned fragments was checked by sequencing. Linearized template DNA was column purified (PCR purification kit, Invitrogen), resuspended in diethyl pyrocarbonate (DEPC)-treated H_2O at a concentration of 1 µg/µl, and stored at -20°C. For both probes *in vitro* transcription was carried out for 3 h at 37°C in a total volume of 20 µl containing 2 µg of linearized plasmid with inserts of desired genes *Drd2* or *Cre recombinase*. Restriction enzymes (New England Biolabs) used for linearization and RNA polymerases used for each probe were as described[7]: *Cre recombinase* antisense: EcoRI, T3; *Cre recombinase* sense: XhoI, T7; *Drd2* antisense: BamHI, T3; Drd2 sense: Eco RI, T7. Pretreatment, hybridization and visualization of signals in fluorescent *in situ* hybridization procedure was carried out as described[8]. Digoxigenin labeled *Drd2*riboprobe was used at a final concentration of 1000 ng/ml hybridization mix, FITC-labeled *Cre recominase* riboprobe at 800 ng/ml.

Supplementary Notes:

Glu-CB1-KO mice display resilience to food addiction

Two additional phenotypic traits considered as a factors of vulnerability to addiction, impulsivity and sensitivity to aversive associative learning, were also evaluated. First, the impulsivity was measured by the inability to stop an action once initiated (responding during the time-out period after each pellet delivery, 10 sec). Glu-CB1-KO mice showed significantly less impulsivity than WT mice only in the late period (U Mann-Whitney, $P<0.01$, Supplementary Figure 1e). Secondly, the aversive associative learning was tested by the ability of the shock-associated cue to suppress pellets seeking the day after the shock-test. Here, in both early and late periods, Glu-CB1-KO mice showed a significantly increased learning with high suppression of food seeking compared to WT mice (U Mann-Whitney, $P<0.01$, Supplementary Figure 1f).

Inhibition of glutamatergic PL neurons promotes food addiction-like behavior

Based on our above observations of decreased development of food addiction in Glu-CB1-KO and increased excitatory transmission in mPFC of these mutants, we hypothesized that on the other hand hypoactivity of glutamatergic transmission in mPFC would promote addictive-like behavior in WT mice when exposed to the palatable food addiction model. To this end, we used a chemogenetic approach to selectively reduce the activity of all the glutamatergic neurons in the PL. We selectively expressed the hM4Di-DREADD in glutamatergic neurons by bilateral injections of a Cre-dependent AAV expressing hM4Di-DREADD (AAV8-hSyn-DIO-hM4D(Gi)-mCherry) into the PL of Nex-Cre mice (Supplementary Figure 4a). Nex-Cre mice express the Cre recombinase specifically in dorsal

telencephalic glutamatergic neurons. Monitoring of mCherry expression allowed to verify injection sites (Supplementary Figure 4b). Next, we aimed at validating our approach by using whole-cell current clamp recordings in L5 of visually identified hM4Di-mCherry-expressing PL neurons in the presence of the selective exogenous ligand clozapine-N-oxide (CNO). We observed reduced excitability of identified hM4Di-expressing PL glutamatergic neurons. CNO application blocked current-evoked action potential firing caused by a decreased membrane resistance (paired t-test, $P<0.05$, Supplementary Figure 4c and Wilcoxon test, $P<0.05$, Supplementary Figure 4d). No significant differences in the firing rate, membrane resistance nor in rheobase were found when CNO was applied in mPFC slices of mice not expressing the hM4Di receptors, suggesting that these CNO-induced effects were selectively mediated by hM4Di receptor activation (Fig. 3d, Supplementary Figure 6b-d).

Therefore, we expected to induce a vulnerable phenotype in those mice expressing inhibitory DREADD in PL when chronically induced hypoactivity of excitatory glutamate transmission using CNO minipumps, leading to the development of food addiction already in the early training period. We trained Nex-Cre mice (n=27) to self-administer chocolate-flavored pellets in the operant chambers under FR1 (2 sessions) and FR5 (3 sessions) schedule of reinforcement before AAV injection and under FR5 (4 sessions) after injection to recover the basal levels of responding (Supplementary Figure 4e). Then, an osmotic minipump filled with CNO (n=14) or saline (n=13) was subcutaneously implanted in the back of each mouse. During the chronic CNO exposure (4 weeks, 0.25 µl/h) with the subsequent inhibition of the glutamatergic PL neurons, mice underwent FR5 sessions for four weeks, and the three food addiction-like criteria were evaluated in the last week. No significant differences between CNO and saline treated mice without inhibitory DREADD expression were found in operant

responding (Supplementary Figure 5a-d), discarding unspecific effects of CNO. In addition, no effect of CNO was found in other parameters, such as body weight, food intake and locomotor activity in mice expressing the inhibitory DREADD (Supplementary Figure 5e-g). Nex-Cre mice expressing hM4Di receptor activated chronically by CNO showed the same number of reinforcers obtained in the daily sessions of the operant conditioning maintained by chocolate-flavored pellets compared to saline treated animals (Supplementary Figure 4f). With regards to the three addiction-like criteria, no differences were obtained in persistence to response, motivation or compulsivity between CNO and saline treated animals (Supplementary Figure 4g-i). Even so, when analyzing the distribution of the individual values, 60% of hM4Di expressing mice were above or equal to the 75th percentile threshold of the control group in motivation and compulsivity criteria. In agreement, 42.8% of mice with the inhibition of glutamatergic PL neurons accomplished the criteria of addiction as compared to 15.4% of saline treated mice (chi-square, $P < 0.01$, Supplementary Figure 4j), suggesting that a decreased excitability of glutamatergic transmission in PL neurons which most likely project to other distinct brain areas is involved in the development of this addictive behavior towards highly palatable food. Positive correlations showed that the intensity of the three food addiction-like criteria was proportional to the number of criteria obtained by the mouse in CNO group and in the persistence to response in saline group (Supplementary Figure 4k-m and the classification od addicted mice showed higher values in both saline and CNO treated mice (Supplementary Figure 5 h-j).

Quantification of cells co-expressing *Drd2* and *Cre* mRNA

For quantification of cells co-expressing *Drd2* and *Cre* mRNA in PL, we counted about 500 cells each on sections from AAV-retrograde-Cre (AAVrg-pmSyn-EBFP-Cre) injected animals. Sections were hybridized with riboprobes for *Drd2* and *Cre*. There were less *Cre*-positive than *Drd2*-positive cells, showing that the injected *Cre*-expressing virus into NAc targeted the region of interest, i.e., PL, but not all *Drd2* expressing cells in PL.

Supplementary References

1. Bellocchio, L. *et al.* Bimodal control of stimulated food intake by the endocannabinoid system. *Nat. Neurosci.* **13**, 281–3 (2010).

2. Martín-García, E. *et al.* Differential Control of Cocaine Self-Administration by GABAergic and Glutamatergic CB1 Cannabinoid Receptors. *Neuropsychopharmacology* **41**, 2192–2205 (2016).

3. Monory, K. *et al.* The endocannabinoid system controls key epileptogenic circuits in the hippocampus. *Neuron* **51**, 455–466 (2006).

4. Marsicano, G. *et al.* The endogenous cannabinoid system controls extinction of aversive memories. *Nature* **418**, 530–4 (2002).

5. Goebbels, S. *et al.* Genetic targeting of principal neurons in neocortex and hippocampus of NEX-Cre mice. *genesis* **44**, 611–621 (2006).

6. Koob, G. F. & Volkow, N. D. Neurocircuitry of addiction. *Neuropsychopharmacology* **35**, 217–38 (2010).

7. Hermann, H., Marsicano, G. & Lutz, B. Coexpression of the cannabinoid receptor type 1 with dopamine and serotonin receptors in distinct neuronal subpopulations of the adult mouse forebrain. *Neuroscience* **109**, 451–460 (2002).

8. Zimmermann, T. *et al.* Neural stem cell lineage-specific cannabinoid type-1 receptor regulates neurogenesis and plasticity in the adult mouse hippocampus. *Cereb. Cortex* **28**, 4454–4471 (2018).
