# Supplementary material for: A specific prelimbic-nucleus accumbens pathway controls resilience versus vulnerability to food addiction
Source: Nat Commun. 2020 Feb 7;11:782. doi: 10.1038/s41467-020-14458-y (PMC7005839; doi:10.1038/s41467-020-14458-y)
Supplement: Supplementary file 3 — Reporting Summary [file 41467_2020_14458_MOESM3_ESM.pdf]

nature research

Corresponding author(s): Rafael Maldonado

Last updated by author(s): Dec 16, 2019

Reporting Summary

Nature Research wishes to improve the reproducibility of the work that we publish. This form provides structure for consistency and transparency in reporting. For further information on Nature Research policies, see Authors & Referees and the Editorial Policy Checklist.

Statistics

For all statistical analyses, confirm that the following items are present in the figure legend, table legend, main text, or Methods section.

n/a	Confirmed	
☐	☒	The exact sample size (n) for each experimental group/condition, given as a discrete number and unit of measurement
☐	☒	A statement on whether measurements were taken from distinct samples or whether the same sample was measured repeatedly
☐	☒	The statistical test(s) used AND whether they are one- or two-sided<br>*Only common tests should be described solely by name; describe more complex techniques in the Methods section.*
☒	☐	A description of all covariates tested
☐	☒	A description of any assumptions or corrections, such as tests of normality and adjustment for multiple comparisons
☐	☒	A full description of the statistical parameters including central tendency (e.g. means) or other basic estimates (e.g. regression coefficient) AND variation (e.g. standard deviation) or associated estimates of uncertainty (e.g. confidence intervals)
☐	☒	For null hypothesis testing, the test statistic (e.g. F, t, r) with confidence intervals, effect sizes, degrees of freedom and P value noted<br>*Give P values as exact values whenever suitable.*
☒	☐	For Bayesian analysis, information on the choice of priors and Markov chain Monte Carlo settings
☒	☐	For hierarchical and complex designs, identification of the appropriate level for tests and full reporting of outcomes
☒	☐	Estimates of effect sizes (e.g. Cohen's d, Pearson's r), indicating how they were calculated

Our web collection on statistics for biologists contains articles on many of the points above.

Software and code

Policy information about availability of computer code

Data collection	For operant conditioning data MedPC-IV was used. For general data collection Microsoft Excel 2016 was used.
Data analysis	SPSS (IBM, version 25, Inc., Chicago, IL, USA) software was used to analyze the data.

For manuscripts utilizing custom algorithms or software that are central to the research but not yet described in published literature, software must be made available to editors/reviewers. We strongly encourage code deposition in a community repository (e.g. GitHub). See the Nature Research guidelines for submitting code & software for further information.

Data

Policy information about availability of data

All manuscripts must include a data availability statement. This statement should provide the following information, where applicable:

- Accession codes, unique identifiers, or web links for publicly available datasets
- A list of figures that have associated raw data
- A description of any restrictions on data availability

Individual data points are graphed in main and supplementary figures. All relevant data that support this study are available from the corresponding author to any interested researcher upon reasonable request and at https://doi.org/10.6084/m9.figshare.11366015. The RNA sequencing data used in this study is available at GEO under accession number GSE139482.

Field-specific reporting

Please select the one below that is the best fit for your research. If you are not sure, read the appropriate sections before making your selection.

☒ Life sciences ☐ Behavioural & social sciences ☐ Ecological, evolutionary & environmental sciences

For a reference copy of the document with all sections, see nature.com/documents/nr-reporting-summary-flat.pdf

Life sciences study design

All studies must disclose on these points even when the disclosure is negative.

Sample size	The sample sizes were calculated based in previous publications performed in our laboratory and on the power analysis. See statistical section for details.
Data exclusions	Animals that responded less than 25% of all FR5 sessions and did not achieve the acquisition criteria were excluded from the operant conditioning mantained by chocolate-flavored pellets: Glu-CB1-KO (14.7%), WT (6.7%).
Replication	Behavioral, immunohistochemical and RT-PCR experiments included replicated experiments to provide compelling evidence of the results.
Randomization	Mice were randomly allocated in their experimental groups, unless they were of a specific genotype and the genotype was one of the between-subject factors of the study.
Blinding	Experiments were performed under blind conditions.

Reporting for specific materials, systems and methods

We require information from authors about some types of materials, experimental systems and methods used in many studies. Here, indicate whether each material, system or method listed is relevant to your study. If you are not sure if a list item applies to your research, read the appropriate section before selecting a response.

Materials & experimental systems

n/a	Involved in the study	
☐	☒	Antibodies
☒	☐	Eukaryotic cell lines
☒	☐	Palaeontology
☐	☒	Animals and other organisms
☒	☐	Human research participants
☒	☐	Clinical data

Methods

n/a	Involved in the study	
☒	☐	ChIP-seq
☒	☐	Flow cytometry
☒	☐	MRI-based neuroimaging

Antibodies

Antibodies used	anti-Cre recombinase (1:500, mouse, MAB3120, Merck Millipore), anti-D2R (1:1000, rabbit, D2R-Rb-Af96, Frontier Institute), anti-mVenus/GFP (1:1000, chicken, ab13970, Abcam) or anti-GFP (1:500, rabbit, GTX20290, GeneTex)
Validation	Each primary antibody was validated by the manufacturer. Several references are provided in the manufacturer's website. We have also validated the efficacy of these primary antibodies during the immunohistochemistry by doing reagent controls without the primary antibody to exclude experimental artifacts.

Animals and other organisms

Policy information about studies involving animals; ARRIVE guidelines recommended for reporting animal research

Laboratory animals	We used Glu-CB1-KO mice (CB1 floxed/floxed; Nex-Cre/+ mice), lacking CB1R in dorsal telencephalic glutamatergic neurons, and their wild-type (WT) littermates in C57BL/6N background (Johannes Gutenberg University Mainz, Germany). Secondly, we used Nex-Cre/+ mice expressing Cre recombinase in dorsal telencephalic glutamatergic neurons (Johannes Gutenberg University Mainz, Germany). Finally, we used WT JAX™ C57BL/6J (C57BL/6J) mice purchased from Charles River (France).
Wild animals	The study did not involve wild animals.
Field-collected samples	The study did not involved samples collected from the field.
Ethics oversight	All experimental protocols were performed in accordance with the guidelines of the European Communities Council Directive 2010/63/EU and approved by the local ethical committee (Comitè Ètic d'Experimentació Animal-Parc de Recerca Biomèdica de

Barcelona, CEEA-PRBB, agreement N°9687). In agreement, maximal efforts were made to reduce the suffering and the number of mice used.

Note that full information on the approval of the study protocol must also be provided in the manuscript.
